# Molecular Characterization of Magnesium Chelatase in Soybean [*Glycine max* (L.) Merr.]

**DOI:** 10.3389/fpls.2018.00720

**Published:** 2018-06-19

**Authors:** Dan Zhang, Enjie Chang, Xiaoxia Yu, Yonghuan Chen, Qinshuai Yang, Yanting Cao, Xiukun Li, Yuhua Wang, Aigen Fu, Min Xu

**Affiliations:** Chinese Education Ministry's Key Laboratory of Western Resources and Modern Biotechnology, Key Laboratory of Biotechnology Shaanxi Province, College of Life Sciences, Northwest University, Xi'an, China

**Keywords:** *Glycine max*, soybean, chlorophyll synthesis, magnesium chelatase, GmChlI, GmChlD, GmChlH

## Abstract

Soybean (*Glycine max*) seed yields rely on the efficiency of photosynthesis, which is poorly understood in soybean. Chlorophyll, the major light harvesting pigment, is crucial for chloroplast biogenesis and photosynthesis. Magnesium chelatase catalyzes the insertion of Mg^2+^ into protoporphyrin IX in the first committed and key regulatory step of chlorophyll biosynthesis. It consists of three types of subunits, ChlI, ChlD, and ChlH. To gain a better knowledge of chlorophyll biosynthesis in soybean, we analyzed soybean Mg-chelatase subunits and their encoding genes. Soybean genome harbors 4 *GmChlI* genes, 2 *GmChlD* genes, and 3 *GmChlH* genes, likely evolved from two rounds of gene duplication events. The qRT-PCR analysis revealed that *GmChlI, GmChlD*, and *GmChlH* genes predominantly expressed in photosynthetic tissues, but the expression levels among paralogs are different. In silicon promoter analyses revealed these genes harbor different *cis*-regulatory elements in their promoter regions, suggesting they could differentially respond to various environmental and developmental signals. Subcellular localization analyses illustrated that GmChlI, GmChlD, and GmChlH isoforms are all localized in chloroplast, consistent with their functions. Yeast two hybrid and bimolecular fluorescence complementation (BiFC) assays showed each isoform has a potential to be assembled into the Mg-chelatase holocomplex. We expressed each GmChlI, GmChlD, and GmChlH isoform in *Arabidopsis* corresponding mutants, and results showed that 4 GmChlI and 2 GmChlD isoforms and GmChlH1 could rescue the severe phenotype of *Arabidopsis* mutants, indicating that they maintain normal biochemical functions *in vivo*. However, GmChlH2 and GmChlH3 could not completely rescue the chlorotic phenotype of *Arabidopsis gun5-2* mutant, suggesting that the functions of these two proteins could be different from GmChlH1. Considering the differences shown on primary sequences, biochemical functions, and gene expression profiles, we conclude that the paralogs of each soybean Mg-chelatase subunit have diverged more or less during evolution. Soybean could have developed a complex regulatory mechanism to control chlorophyll content to adapt to different developmental and environmental situations.

## Introduction

Soybean [*Glycine max* (L.) Merr.] is one of the most economically important crops as the main sources of plant protein and vegetable oil (Dornbos and Mullen, 1992; Ainsworth et al., 2012). Global soybean yield has been steadily increased over the past century attributed to improved cultivars and agronomy management, but average soybean yield is still away from reaching a plateau (Egli, 2008; Masuda and Goldsmith, 2009). In order to meet the growing need resulting from a fast expanding population and limited agricultural land, soybean yield and quality must improve at a higher speed than before. Multiple agricultural approaches can be exploited to achieve this goal, such as improving photosynthetic efficiency, optimizing utilization of carbon, increasing the efficiency of nitrogen fixation, and adjusting developmental process (Ainsworth et al., 2012; Natarajan et al., 2013; Koester et al., 2014).

Previous reports have shown that soybean seed yields are positively correlated to the increase in light interception, energy conversion, and partitioning efficiencies (Zhu et al., 2010; Koester et al., 2014), suggesting that improving photosynthetic efficiency could be a promising method to raise soybean production in the future. However, little is known about the molecular base of photosynthesis in soybean up to date, because soybean is not an ideal material to perform plant physiology study at molecular biology level. Soybean is a diploid (2*n* = 40) crop evolved from a recent tetraploid ancestor, possessing a 1.1-gigabase genome with approximate 50,000 genes, in which 75% are duplicated (Shoemaker et al., 2006; Schlueter et al., 2007). The complexity of genome and shortage of molecular biology tools are big obstacles to dissect soybean photosynthesis in detail.

Chlorophyll content of plant leaves is the major limiting factor for the efficiency of photosynthesis (Chen and Blankenship, 2011). Chlorophyll is the main light harvesting and energy converting pigment for photosynthesis (Croce and van Amerongen, 2014). It is composed of a chlorophyllide moiety and an isoprenoid phytol tail, which are generated through the tetrapyrrole biosynthetic pathway and methylerythritol phosphate (MEP) metabolic pathway, respectively (Masuda and Fujita, 2008; Kim et al., 2013; Croce and van Amerongen, 2014). The genes and corresponding enzymes involved in the chlorophyll biosynthesis pathway have been well-characterized in model photosynthetic organisms; however, the regulatory mechanisms of the pathway are only recently studied and not fully understood (Masuda and Fujita, 2008; Brzezowski et al., 2015).

In the first committed step of chlorophyll biosynthesis, magnesium chelatase (E.C.6.6.1.1, Mg-chelatase) inserts a magnesium ion (Mg^2+^) into protoporphyrin IX to generate Mg-protoporphyrin IX (Masuda, 2008). Mg-chelatase is a highly conserved polymeric enzyme composed of 3 distinct subunits, ChlI, ChlD, and ChlH, with an approximate molecular weight of 40, 70, and 140 kDa, respectively (Walker and Willows, 1997; Sirijovski et al., 2006). The ChlI subunit belongs to the large AAA^+^ family (ATPase Associated with various cellular Activities), contains typical ATP-binding motifs in sequence like Walker A and Walker B, and is responsible for ATP hydrolysis (Hansson et al., 2002; Lake et al., 2004). The ChlD subunit has an AAA^+^ module at its N-terminus, an integrin I domain at the C-terminus, and an acidic proline-rich region in between; however, no ATPase activity is detected in ChlD (Gräfe et al., 1999; Fodje et al., 2001). The ChlH subunit is the porphyrin binding and catalytic subunit responsible for the insertion of Mg^2+^ into protoporphyrin IX (Jensen et al., 1998; Karger et al., 2001). It is composed of six domains (I–VI), and an internal Proto-binding pocket is located at the interface between domain III and V possibly functioning in engulfing a tetrapyrrole ligand (Chen et al., 2015).

The magnesium chelation reaction has been postulated to proceed in two steps. During initial activation step, 6 ChlI and 6 ChlD subunits are assembled into a two-tiered hexameric ring in the presence of ATP, and meanwhile ChlH are activated by binding Mg^2+^ and protoporphyrin IX. Next, the activated ChlH docks to the ATP-I-D complex to form Mg-chelatase holoenzyme, catalyzing Mg^2+^ chelation into protoporphyrin IX in an ATP hydrolysis-dependent manner (Sirijovski et al., 2006; Zhang et al., 2006; Masuda, 2008).

The Chlorophyll biosynthesis has to be tightly controlled to fit the requirement of chloroplast biogenesis, or to maintain proper function of photosynthetic machineries. Most of chlorophyll intermediates are strong photosensitizers, and they will accumulate and further produce reactive oxygen species to damage cells if the regulation of chlorophyll biogenesis is impaired (Masuda and Fujita, 2008; Stephenson and Terry, 2008). Magnesium chelation, the branch point between the heme and chlorophyll biosynthetic pathways, is the major regulatory point of the chlorophyll biosynthesis pathway. It is well-known that Mg-chelatase is tightly controlled by the light signaling pathway at transcriptional level during thylakoid biogenesis in young chloroplasts, and is also regulated by a diurnal cycle and photosynthetic electron transport at both transcriptional and post-transcriptional levels in mature chloroplasts (Masuda, 2008).

Several Mg-chelatase impaired mutant plants have been identified in soybean, and they showed chlorophyll deficient in heterozygous and homozygous mutant plants (Palmer et al., 1989; Campbell et al., 2015). In one Mg-chelatase impaired mutant, the chlorophyll amount of heterozygous plants reduces to 50% of the wild-type level, however the yield remains a similar level compared to wild-type plants (Slattery et al., 2017; Walker et al., 2017), or even is higher than wild-type plants (Pettigrew et al., 1989). It suggests a possibility to improve photosynthesis capacity and seed yield in soybean by means of manipulating chlorophyll content. As a key enzyme and major regulatory point of chlorophyll biogenesis, Mg-chelatase might be a primary target to engineer at molecular level.

One hurdle to regulate the activity of Mg-chelatase with molecular biology tools is that its molecular features and regulatory mechanisms are not well-understood in soybean. The complete genome sequence of soybean was released in 2010 (Schmutz et al., 2010). Along with improved gene transformation tools in soybean, it provides an opportunity to dissect the photosynthesis process in detail, and further to manipulate some key components to improve photosynthesis efficiency. To gain a better knowledge of chlorophyll biogenesis in soybean, we take advantage of soybean genome data and molecular biology tools to examine all Mg-chelatase subunits at genomic, transcriptional, and protein levels. We think this knowledge will help us to better understand this key enzyme in soybean chlorophyll biosynthesis pathway; and it could lay a foundation to manipulate soybean Mg-chelatase for further improving photosynthesis efficiency.

## Materials and methods

### Primers

All the primers used in present study are listed in Supplementary Table S1.

### Plant materials and growth conditions

Soybean cultivar Williams 82 (Wm82) and *Nicotiana benthamiana* plants were grown in green house at 26°C with a photoperiod of 16 h light/8 h dark cycle at a photosynthetic flux of 140 μmol^.^m^−2.^s^−1^.

Five *Arabidopsis thaliana* lines were used in this study, including wild-type Columbia (Col-0), Lansberg *erecta* (L*er-0*), and three T-DNA insertion mutant lines, *chli1* (Sail_230_D11, L*er-0* background), *chld-2* (009D11, Col-0 background), and *gun5-2* (CS806665, Col-0 background). The *chli1, chld-2*, and *gun5-2* mutants were obtained from Hsou-min Li (Institute of Molecular Biology, Academia Sinica, Taipei), GABI-Kat (https://www.gabi-kat.de), and Fang-Qing Guo (Institute of Plant Physiology & Ecology, Shanghai, China), respectively. All these T-DNA insertion lines were confirmed with the primers listed in Supplementary Table S1.

*Arabidopsis* plants were grown in soil or on half-strength Murashige and Skoog (1/2 MS) medium (PhytoTech, USA) containing 1% sucrose at 23°C under a 16 h light/8 h dark cycle with an illumination of 100 μmol^.^m^−2.^s^−1^.

### Sequence retrieval and bioinformatics analysis

A search for soybean genes encoding Mg-chelatase I subunit (GmChlI), D subunit (GmChlD), and H subunit (GmChlH) was performed by using the BLASTN program against the soybean genome (https://soybase.org) with the coding sequences (CDSs) of *Arabidopsis* Mg-chelatase subunits I1 (AtChlI1), D (AtChlD), and H (AtChlH) (Mochizuki et al., 2001; Huang and Li, 2009; Du et al., 2012). The cDNA fragments of retrieved *GmChlI*s, *GmChlD*s, and *GmChlH*s were amplified by RT-PCR and further sequenced. A primary comparison between the deduced amino acid sequences of each subunit and their corresponding homologs from *Arabidopsis* and *Synechocystis* sp. PCC6803 (Ssp. PCC6803) was conducted with multiple sequence alignment program ClusterW2.

A further phylogenetic analysis was performed to elucidate the evolutionary history of GmChlI, GmChlD, and GmChlH. The amino acid sequences of ChlI, ChlD, and ChlH from 11 species in addition to soybean were obtained from NCBI database (https://www.ncbi.nlm.nih.gov), including 2 legume species (*Phaseolus vulgaris* and *Cajanus cajan*), 4 dicots other than legumes (*A. thaliana, Gossypium arboreum, Populus trichocarpa*, and *Vitis vinifera*), 4 monocots (*Oryza sativa, Zea mays, Brachypodium distachyon*, and *Sorghom bicolor*), and 1 cyanobacterium (Ssp. PCC6803). Sequences were aligned with the ClustalW2 program, and phylogenetic trees were constructed by MEGA version 6.0 (Tamura et al., 2013) based on the Neighbor-Joining method.

To investigate the potential regulation of gene expression, 1,500 bp sequences immediately upstream of the translation start codon of *GmChlI, GmChlD*, and *GmChlH* were subject to the analysis of putative *cis*-acting regulatory elements by using the PlantCARE online program (http://bioinformatics.psb.ugent.be/webtools/plantcare/html/, Lescot et al., 2002).

### RNA extraction, RT-PCR, and real-time qRT-PCR

Total RNA was extracted from desired soybean tissues by RNAPrep pure plant kit (TIANGEN, China) following the manufacturer's instruction. For regular RT-PCR, RNA from leaf tissue was used. For qRT-PCR analysis, RNAs were extracted from tissues at different developmental stages. The roots and cotyledons were harvested from 1-week old seedlings. The stems, young trifoliate leaves, and flowers were sampled from flowering plants at R2 stage (~45-day old). Young pods (~4 cm long) and immature seeds (~1 cm in length) from 4 cm long pods were sampled from plants at R5 stage (65~70-day old).

Total RNA was treated with Turbo DNA-free kit (Invitrogen, USA) to remove genomic DNA contamination before reverse transcription. One microgram of DNA-free RNA was reverse transcribed to 1st strand cDNA using the PrimeScript II 1st strand cDNA Synthesis Kit (Takara, Japan) according to the manufacturer's protocol. The full-length coding sequences of *GmChlI*s, *GmChlD*s, and *GmChlH*s were amplified from Wm82 leave cDNA by PCR with PrimerSTAR Max DNA polymerase (Takara, Japan). PCR was run with an initial pre-denaturation of 98°C for 30 s, followed by 30 cycles of 98°C for 15 s, 60°C for 15 s, and 72°C for 2 min. The PCR products were cloned into pMD18T vector (Takara, Japan) for sequencing.

For qRT-PCR analysis, the 1st strand cDNA was diluted 20 times, and 1 μl was used in each real time quantitative PCR. Quantitative PCR was performed on BioRad CFX96 with 2x SYBR Green qPCR Master Mix (Roche, Switzerland) in 20 μl reaction. PCR was run with the following program: 10 min in 95°C, followed by 40 cycles of 10 s at 95°C and 30 s at 63°C, then increasing up to 95°C at an increment of 0.5°C degree per min. The cycle threshold value was calculated by the CFX Manager Software version 3.0 (Bio-Rad, USA). Relative gene expression level was calculated by using the 2^−ΔCt^ method. All data were normalized against the expression level of the soybean *actin* gene (*Glyma18g290800*). For each sample, three replicates were performed.

### Subcellular localization and bimolecular fluorescence complementation (BiFC)

Vectors pSPYNE and pSPYCE (Waadt et al., 2008) carrying the CaMV 35S promoter-driven N- and C-terminal half of YFP, respectively, were used for BiFC assay. Vector pSPY-GFP for localization is generated by replacing *YFP*_*N*_ in pSPYNE with *GFP* gene. Coding sequences of *GmChlI*s, *GmChlD*s, and *GmChlH*s were cloned into pSPY-GFP, pSPYNE, and pSPYCE, in-frame fused to the N-terminus of the corresponding tags.

For subcellular localization, *Agrobacterium tumefaciens* (GV3101) carrying vectors expressing GmChlI-GFP, GmChlD-GFP, and GmChlH-GFP were co-infiltrated with the p19 strain into *N. benthamiana* leaves for transient expression as described in Waadt et al. (2008). Similarly, for BiFC, pairs of YFP_N_ and YFP_C_ fusion proteins were transiently co-expressed in the leaves of *N. benthamiana*. GFP and reconstructed YFP fluorescence were observed and imaged 2–3 days after infiltration using the Olympus Fluoview FV1000 confocal laser scanning microscope (Olympus, Japan).

### Yeast two hybrid assay

The yeast two-hybrid (Y2H) analysis was performed using the Matchmaker GAL4 Two-Hybrid System 3 according to the supplier's instruction (Clontech, USA). Coding sequences for mature peptides of GmChlIs, GmChlDs, and GmChlHs were cloned into both the bait vector pGBKT7 and the prey vector pGADT7, downstream in frame to the GAL4 DNA binding domain (BD) and the GAL4 activation domain (AD), respectively. Pairs of the resulting BD and AD constructs were co-transformed into yeast strain AH109 according to the manufacturer's instruction. Yeast cells were first grown on synthetic dropout medium missing Leu and Trp, and then the colonies were tested for growth on selective medium lacking Leu, Trp, His, and Ade.

### Gene transformation in *Arabidopsis* and transgenic plant screening

Full length CDSs of *GmChlI*s, *GmChlD*s, and *GmChlH*s tagged with *HA* at C-terminus were cloned into pSPYCE by replacing the *YFP*_*C*_ fragment, and transformed into the corresponding *Arabidopsis* mutants, heterozygous *chli1*/+, *chld-2*/+, and homozygous *gun5-2*, through *Agrobacterium* mediated transformation (Clough and Bent, 1998). Transgenic lines were screened on MS plates against kanamycin. The genotypes at *chli1* or *chld-2* loci were evaluated by PCR in *GmChlI-HA* and *GmChlD-HA* transformants. Genomic DNA for PCR analysis was extracted from leaves using NuClean PlantGen DNA Kit (CWBIO, China). Transgenic plants with homozygous mutant background would be advanced to next generation for further analysis. Three-week old T_3_ plants derived from three independent T_1_ transgenic lines were surveyed for phenotypes and photographed. The lines that did not show a full complementation by the transgene were subject to further examinations of protein and chlorophyll content. The protein level of these transgenic lines was examined by western-blot analysis with HA monoclonal antibody (H9658, Sigma-Aldrich, USA).

### Chlorophyll (Chl) content measurement

The chlorophyll was extracted from leaves by immersing in a 4.5: 4.5: 1 (v/v) mixture of acetone, ethanol, and distilled water for 12 h under dark condition. After extraction, the absorbance was measured at 663 and 645 nm with UV-VIS Double Beam Spectrophotometer (HALO DB-30, Dynamica, UK). Chlorophyll contents (mg per gram of fresh weight tissue, mg/g) were calculated using the following equations (Deng et al., 2014):

Chl_a_ (mg/g) = [(9.978OD_663_-0.99OD_645_) x V(ml)]/(tissue weight (g) x1000)

Chl_b_ (mg/g) = [(21.426OD_645_-4.65OD_663_) x V(ml)]/(tissue weight (g) x1000)

Total Chl (mg/g) = Chl_a_ (mg/g) + Chl_b_ (mg/g)

The experimental result for each line was expressed as mean ± standard deviation (sd) of three replicates. Statistical significance of differences between transgenic lines and corresponding wild type or mutant lines were tested using the two-tailed Student's *t*-test algorithm. *P*-value < 0.05 was regarded as significant.

### Accession number

We deposited all the soybean chelatase genes in this article to the GenBank/EMBL databases under the following accession numbers: *GmChlI1a* (MG679388), *GmChlI1b* (MG696843), *GmChlI2a* (MG696844), *GmChlI2b* (MG696845), *GmChlD1* (MG696846), *GmChlD2* (MG696847), *GmChlH1* (MG696848), *GmChlH2* (MG696849), and *GmChlH3* (MG696850).

## Results and discussion

### Identification of the Mg-chelatase subunits in soybean

We took the first step to undermine genes encoding Mg-chelatase subunits in the soybean genome using *Arabidopsis* Mg-chelatase subunits (AtChlI1, AtChlD, and AtChlH) as query references. After a genome-wide database searching in the soybean genome (https://soybase.org), we found 4 GmChlI, 2 GmChlD, and 3 GmChlH encoding genes. Gene names were assigned to respective loci as following: Glyma13g232500 (*GmChlI1a*), Glyma15g080200 (*GmChlI1b*), Glyma7g204300 (*GmChlI2a*), Glyma13g171800 (*GmChlI2b*), Glyma11g016000 (*GmChlD1*), Glyma1g226700 (*GmChlD2*), Glyma13g13700 (*GmChlH1*), Glyma19g139300 (*GmChlH2*), and Glyma10g097800 (*GmChlH3*). Out of these 9 genes, *GmChlI1a, GmChlI1b*, and *GmChlH1* have been reported previously (Nakayama et al., 1995, 1998; Campbell et al., 2015).

The cDNA of each gene was amplified by RT-PCR from Wm82 leaf tissue and sequenced to determine the right splicing forms. According to the sequencing results, *GmChlI1a, GmChlI1b*, and *GmChlI2b* encode a ~40 kDa polypeptide with 433 amino acid (aa), while *GmChlI2a* encodes a 415-aa polypeptide. Proteins encoded by *GmChlID1* and *GmChlD2* are 751-aa long with molecular weight of ~70 kDa. *GmChlH1* and *GmChlH3* both encode 1385-aa polypeptides with molecular weight of ~140 kDa, while *GmChlH2* encodes a 1384-aa polypeptide. All soybean ChlI, ChlD, and ChlH subunits are similar in molecular mass to their corresponding homologs identified in other species (Figure S1) (Jensen et al., 1996; Sawers et al., 2006; Zhang et al., 2006; Du et al., 2012; Muller et al., 2014).

According to the prediction by TargetP, each soybean Mg-chelatase subunit contains a chloroplastic transit peptide (CTP) at the N-terminus as expected. Sequence comparison of GmChlIs, GmChlDs, and GmChlHs to their orthologs from *A. thaliana* and Ssp. PCC6803 revealed that they are all well-conserved (Figures S1–S3). Four GmChlIs are highly similar, sharing at least 90% amino acid identity, and each of them contains the full set of characteristic motifs in ChlI, including Walker A, Walker B, Sensor I, Arginine fingers (R finger), and Sensor II (Figure S1). GmChlID1 and GmChlD2 share 97% identity on amino acid sequence (Figure S2). All three structural domains of ChlD are highly conserved in GmChlD proteins, including an AAA^+^ like module at the N-terminus, a proline-rich linker region, and an integrin I domain at the C-terminus (Figure S2). GmChlH1 and GmChlH2 share 99% identity on amino acid sequence and both of them share 97% sequence identity with GmChlH3 (Figure S3). The sequences of three GmChlHs are conserved in all six functional domains, especially in domains III, V, and VI that constitute the putative active center (Chen et al., 2015). The residues surrounding the putative tetrapyrrole-binding pocket are invariant compared to SspChlH. Together, the primary structure information indicates that all isoforms of GmChlI, GmChlD, and GmChlH are likely functional *in vivo*.

### Phylogenetic analysis of Mg-chelatase subunits in soybean

**Figure 1:**
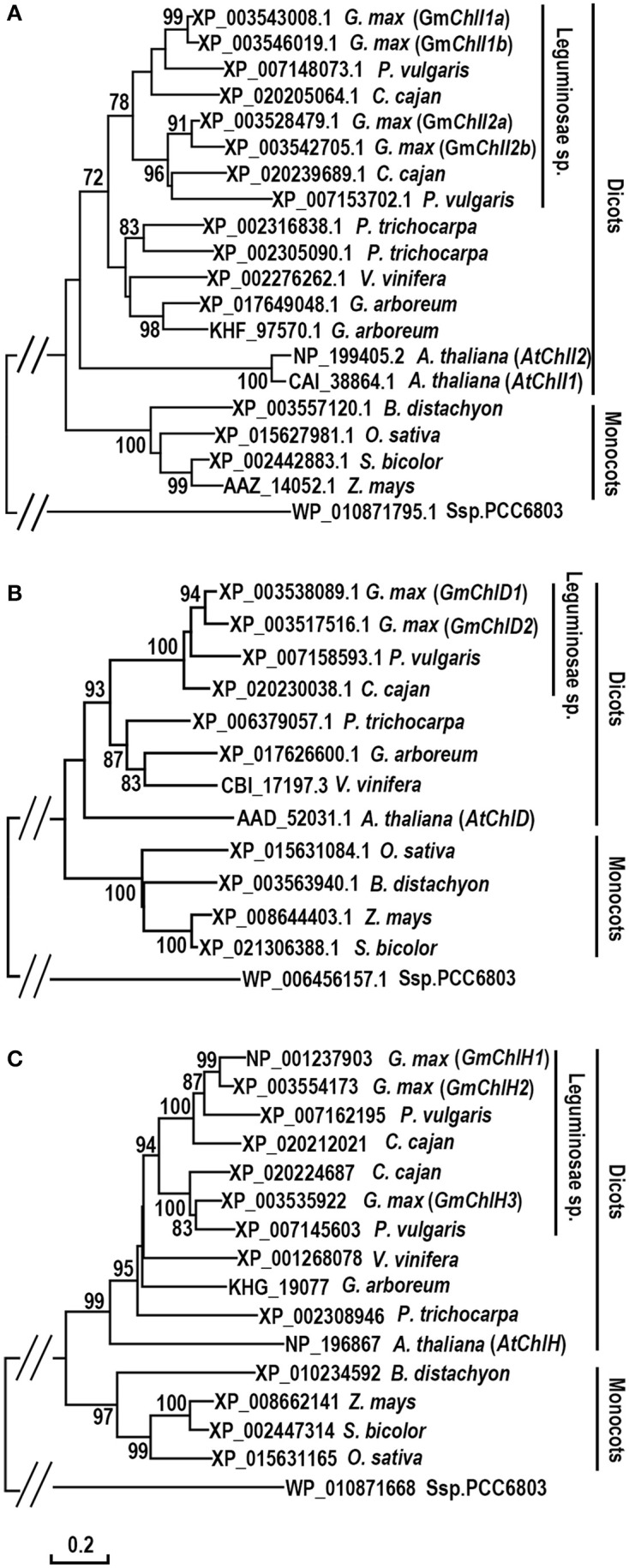
Phylogenetic analysis of ChlI, ChlD, and ChlH subunits of Mg-chelatase**. (A)** Evolutionary relationships of ChlI subunits; **(B)** Evolutionary relationships of ChlD subunits; **(C)** Evolutionary relationships of ChlH subunits. Sequences were taken from *G. max* and 11 species from *cyanobacterium*, monocots, and dicots, of which the sequences of ChlI, ChlD, and ChlH subunits from *A. thaliana* (Du et al., 2012), *O. sativa* (Zhang et al., 2006; Muller et al., 2014), Ssp. PCC6803 (Jensen et al., 1996), and the ChlI subunit from *Z. mays* (Sawers et al., 2006) were published previously. The unrooted phylogenetic trees were constructed using the Neighbor-Joining method and a 1,000 bootstrap resampling value. The bootstrap values >70% are shown next to the branches. The evolutionary distances were computed using the Poisson correction method and are in the units of the number of amino acid substitutions per site. Positions containing gaps and missing data were eliminated. Evolutionary analyses were conducted in MEGA6.

To investigate the evolution history of Mg-chelatase, protein sequences of GmChlI, GmChlD, and GmChlH were subjected to phylogenic analysis along with their orthologs from 11 species including 6 dicotyledons (*P. vulgaris, C. cajan, V. vinifera, G. arboreum, P. trichocarpa, A. thaliana*), 4 monocotyledons (*B. distachyon, Z. mays, S. bicolor, O. sativa*), and 1 cyanobacterium (Ssp. PCC6803). The sequences of ChlIs, ChlDs, and ChlHs are clustered into dicot and monocot clades along with Ssp. PCC6803 as an out-group, and subsequently diverged by family, showing that all the homologs from legume family are sub-grouped together (Figure 1). In legumes, most of species encode 2 ChlIs, 1 ChlD, and 2 ChlHs, in which the ChlI and ChlH subunits are further sub-divided into two clusters, indicating that these two subunits experienced a duplication at the origin of the legumes. Comparatively, soybean genome encodes 4 GmChlIs, 2 GmChlDs, and 3 GmChlHs, indicating that Mg-chelatase genes went through a second round of duplication at the origin of soybean. Therefore, four GmChlIs are separated into two groups, each belonging to one of the legume ChlI clusters. One group contains GmChlI1a and GmChlI1b while the other one contains GmChlI2a and GmChlI2b (Figure 1A). Similarly, three isoforms of GmChlH are divided into two groups as well, with GmChlH1 and GmChlH2 as one group and GmChlH3 as the other group (Figure 1C). The twin copy of *GmChlH3* gene is likely lost after the gene duplication event.

In addition, an interesting history is observed in the evolution of ChlI subunit (Figure 1A). Most dicotyledons encode 2 ChlI isoforms whereas monocotyledons and Ssp. PCC6803 only have 1 ChlI. It is logical to postulate that a duplication event of ChlI would happen near the origin of the dicots. However, the phylogenetic tree shows that ChlIs from the same dicotyledon species or family tend to cluster together indicating that various independent duplications of ChlI subunit happened during the evolution of dicots (Figure 1A).

### Tissue specific expression profiles of *GmChlI*s, *GmChlD*s, and *GmChlH*s

To illustrate whether the paralogs of *GmChlI, GmChlD*, and *GmChlH* genes are diverged in biological function, we analyzed the tissue specific expression profile of each Mg-chelatase gene through real-time quantitative RT-PCR. The transcription levels of *GmChlI*s, *GmChlD*s, and *GmChlH*s were investigated in various tissues, including roots, stems, cotyledons, leaves, flowers, pods, and immature seeds. The results revealed that the tissue expression patterns of all the genes are generally similar, with the highest level in leaves followed by cotyledons, a relative lower level in stems, flowers, and pods, and an almost negligible level in roots.

**Figure 2:**
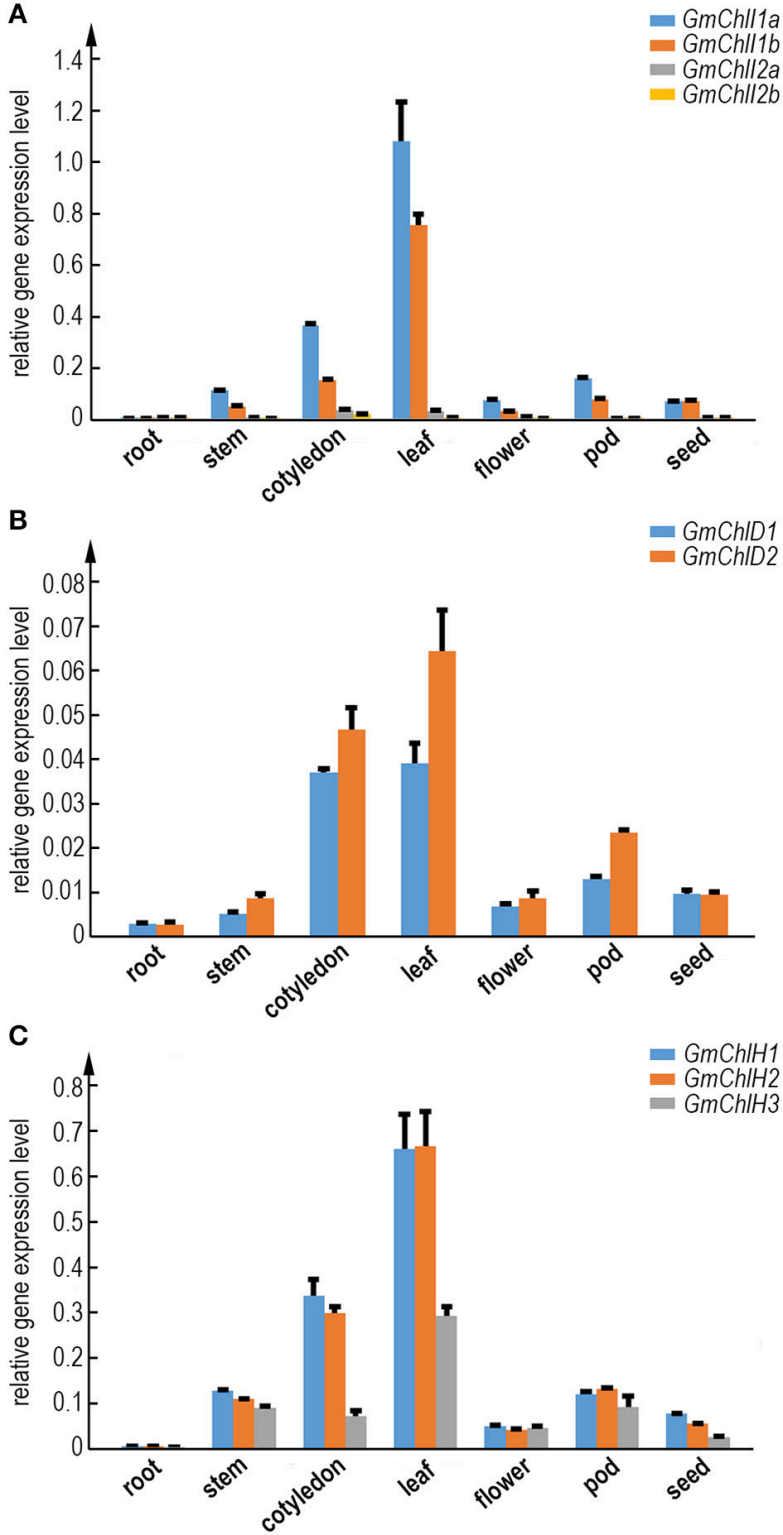
Expression analysis of GmChlIs, GmChlDs, and GmChlHs in various tissues at different growth stage of soybean. Expression profiles of four *GmChlI* genes **(A)**, two *GmChlD* genes **(B)**, and three *GmChlH* genes **(C)** in different tissues are obtained by quantitative RT-PCR. The names of tissues are indicated below the chart. Root and cotyledon were taken from 7-day old seedlings. Stem, trifoliolate leaves, and flowers were taken from ~45-day-old flowering plants. Pods (~4 cm long) and immature seeds (~1 cm in length) were sampled from 65~70-day old plants. Expression level of each gene is normalized to those of actin. Error bars indicate standard deviation (sd) from three technical replicates.

The expression levels between paralogs are different, but the differences between closely related paralogs are smaller compared to distantly related ones (Figures 1, 2). For example, *GmChlI1a* and *GmChlI1b* express much higher than *GmChlI2a* and *GmChlI2b*, which are barely expressed in all examined tissues (Figure 2A). Meanwhile, the expression levels of *GmChlI1a* and *GmChlI2a* are about 2-fold higher compared to *GmChlI1b* and *GmChlI2b*, respectively, in most of the tissues. These data imply that GmChlI1a and GmChlI1b are the major functional Mg-chelatase I subunit in soybean. The expression levels of two *GmChlD* genes were comparable in all tissues, but *GmChlD2* is expressed at a slightly higher level (less than 2 fold) compared to *GmChlD1* (Figure 2B), suggesting both of GmChlD paralogs function in soybean. Among three *GmChlH* genes, the expression of *GmChlH3* is generally lower than the other two especially in leaves and cotyledons, and no significant difference was detected between *GmChlH1* and *GmChlH2* (Figure 2C). Taken together, the transcription levels of *GmChlIs, GmChlDs*, and *GmChlHs* show a positive correlation with the tissue photosynthetic activity, which is consistent to the enzyme function. However, the functions of the paralogs of each subunit are likely diverged with respect to their different expression levels.

### Promoter analyses of *GmChlI*s, *GmChlD*s and *GmChlH*s

We next carried out a promoter motif analyses to further study the potential regulatory mechanisms of *GmChlIs, GmChlDs*, and *GmChlHs*. The 1,500 bp region immediately upstream of start codon ATG of each gene was analyzed with the online program PlantCARE (Lescot et al., 2002). Multiple CAAT boxes and various *cis*-acting regulatory motifs were predicted from the promoter regions of these genes, including light responsive elements (LREs), circadian regulatory elements (CREs), hormone responsive elements (HREs), and defense and stress responsive elements (DSREs) (Tables 1, 2).

**Table 1:** Predicted CAAT boxes and *cis*-elements response to light and circadian in the promoter regions of *GmChlIs, GmChlDs*, and *GmChlHs* genes.

**Motifs**	**Sequence**	***GmChlI1a***	***GmChlI1b***	***GmChlI2a***	***GmChlI2b***	***GmChlD1***	***GmChlD2***	***GmChlH1***	***GmChlH2***	***GmChlH3***
CAAT box^a^	CAAT/CCAAT	−98,−164	−39,−93	−216	−55,−104	−88,−30	−28,−91	−35,−54	−52,−80	−359
		−179,−203	−166,−181		−394	−178,−242	−147,−227	−62, −85	−100,−251	
		−261,−296	−282				−273,−278	−112,−240	−295,−371	
							−315	−353		
**Subtotal**		**6**	**5**	**1**	**3**	**4**	**7**	**7**	**6**	**1**
**Light response**^b^									
AAAC-motif	CAACAAAAACCT	–	–	–	–	–	−579	−386	–	–
ACE	GACACGTATG	−850	−859	–	–	–	–	–	−751	−1150
AE-box	AGAAACAT	−53	−48	−17	−1017	–	−602	−335	−910, −1572	–
AT1-motif	AATTATTTTTTATT	–	−578	–	−695	–	–	–	–	–
ATCT-motif	AATCTGATCG	–	–	–	–	−58	55	−524	−541	–
Box 4	ATTAAT	−184, −682	−186, −540	–	−297,−685	−507,−1034	−595,−1144	–	–	−68, −891
			−589,−908		−982, −1433	−1453	−1477			−1307
Box I	TTTCAAA	−771	−1129	−345,−375	–	–	–	−173, −94	−109,−184	−63, −1302
				−509					−1405	
Box II	CCACGTGGC	–	–	–	–	–	−203	−834	–	−335
CATT–motif	GCATTC	–	–	–	−1441	−748,−752	–	–	–	–
						−1242				
GAG-motif	AGAGAGT	–	–	−173	–	–	–	–	−435	–
GA-motif	AAGGAAGA	–	–	−22,−131	−161	–	–	–	−77	–
GATA-motif	AAGGATAAGG	−964	−611	−121	–	–	–	–	−73	–
G-box	CACGTG	−428	−84	−65	−391	−1321,−1374	−192,−201	−832	−343	−333
GT1-motif	GGTTAA	−538,−854	–	−272	−702	–	−100,−1058	−978	−938	–
Gap-box	AAATGGAGA	–	–	–	–	−1209	–	–	–	–
I-box	GATAAGATA	−964	−995	−123	–	−57	−554	−500, −1418	−517,−1467	−86
MNF1	GTGCCC	–	–	–	–	–	−1248	–	–	–
Sp1	CC(G/A)CCC	−106,−968	−101	−825,−860	−1389	–	−536	–	−124	−514
		−1284								
TCCC-motif	TCTCCCT	–	–	–	–	–	–	−566	–	–
TCT-motif	TCTTAC	–	−619	–	−630,−1145	−707,−1416	–	−46,−738	–	−681,−1180
chs-CMA1a	TTACTTAA	–	–	–	−634	–	–	–	–	–
**Subtotal**		**13**	**13**	**13**	**14**	**13**	**14**	**13**	**15**	**12**
**Circadian response** ^b^									
Circadian	CAAAGATATC	–	−1308	–	–	−921	−1208	−688	−708	−69,−1282

*The positions of the cis-elements with respect to the translation initiation site (+1) are list in the table*.

a *CAAT boxes are predicted from 400 bp upstream promoter region of GmChls*.

b *These cis-elements are predicted from 1.5 kb upstream promoter region of GmChls*.

**Table 2:** Predicted *cis*-elements response to hormones, stresses, and specific tissues in the 1.5 kb promoter regions of *GmChlIs, GmChlDs*, and *GmChlHs* genes.

**Motifs**	**Sequence**	***GmChlI1a***	***GmChlI1b***	***GmChlI2a***	***GmChlI2b***	***GmChlD1***	***GmChlD2***	***GmChlH1***	***GmChlH2***	***GmChlH3***
**HORMONE AND STRESS RESPONSE**										
AuxRR-core (auxin, growth)	GGTCCAT	–	–	–	−266	–	–	−1120	−168	–
TGA-element (auxin, growth)	AACGAC	–	–	−259	–	−15	–	–	−1158	–
GARE-motif (GA, growth)	(A/C)AACAGA	–	–	−1213	−411,−934	–	–	−488, −1443	−505	−58
P-box (GA, growth)	CCTTTTG	−636	−736, −855	−925, −1250	–	–	–	–	–	–
ABRE (ABA, stress)	CACGTG	−428	–	−65	–	−1377	−203	−334	−343	−335
ERE (ethylene,stress)	ATTTCAAA	–	–	−376, −509	–	–	–	–	–	–
TCA-element (SA, stress)	GAGAAGAATA	−145	−627	−1380	−88, −258	−1172	–	−1062	−1021	−48, −820, −910
CGTCA-motif (MeJA, stress)	CGTCA	–	−82	–	–	–	−613	–	–	–
HSE (heat)	AAAAAATTTC	–	−492, −900, −1155	−341, −381, −516, −1010	−288, −584	−863	−189	−619, −944, −1409	−636	−651, −1197
LTR (low temperature)	CCGAAA	–	–	–	–	−1388	–	−546	−563	−497, −947
MBS (drought)	TAACTG	−1276, −1303	–	−1205	−903,−1158, −1308	−807	–	−914	−873	−175, −310, −1262
TC-rich repeats (Pathogen)	ATTTTCTTCA	−594	−623, −868, −897, −1087	−343, −802	−741	–	–	−63, −990	−410, −950, −1189, −1211	−681
**Subtotal**		**6**	**11**	**15**	**11**	**6**	**3**	**12**	**12**	**13**
**TISSUE SPECIFIC**										
GCN_motif (Endosperm)	TGTGTCA	−1073	–	–	–	–	–	–	−1165	−358, −865
Skn-1_motif (Endosperm)	GTCAT	−400, −483	−1186, −1220	–	−242, −393, −761, −851	−636, −621, −716, −930, −997	−1191, −1460	−398, −798, −1084, −1305	−450, −744	−238, −349, −884, −866
CAT-box (Meristem)	GCCACT	−1096, −1202	–	–	–	−1343, −1354	–	–	–	–
HD-Zip1 (leaf)	CAATGATTGCCAG	–	–	–	–	–	−279	–	–	–
**Subtotal**		**5**	**2**	**–**	**4**	**7**	**3**	**4**	**3**	**6**

*The positions of the cis-elements with respect to the translation initiation site (+1) are list in the table*.

The CAAT box is a proximal promoter element recognized by CAAT-box binding transcription factors and is important for the sufficient transcription of the downstream gene (Bi et al., 1997). We counted the predicted CAAT boxes in the 400 bp region upstream of Mg-chelatase genes, revealing a consistency with their transcription levels detected by qRT-PCR. The paralogs with more CAAT boxes generally show a higher expression compared to the ones with less CAAT boxes in most of tissues (Figure 2 and Table 1).

Among *cis*-acting regulatory motifs, it is not surprised that LREs are the most abundant motifs in promoter regions of *GmChlI*s, *GmChlD*s, and *GmChlH*s (Table 1). Light is the main environmental factor regulating chlorophyll biosynthesis; it is required for massive expression of Mg-chelatase genes (Papenbrock et al., 1999; Winter et al., 2007; Stephenson and Terry, 2008). The total number of LREs is similar among 9 promoters, ranged from 12 to 15, in which G-box is commonly present (Table 1). G-box is the bind site of many transcription factors in light signaling pathway, such as ELONGATED HYPOCOTYL5 (HY5) and PHYTOCHROME INTERACTING FACTOR proteins (PIFs) (Toledo-Ortiz et al., 2014). Other than G-box, light responsive motifs are different across promoters, suggesting there is a difference in fine regulation of soybean Mg-chelatase genes responsive to light.

Several reports show that the expression of *ChlH* exhibits a diurnal oscillation pattern when plants are grown in light-dark regime whereas the expression of *ChlI* and *ChlD* displays less or no variation under the same situation (Gibson et al., 1996; Jensen et al., 1996; Papenbrock et al., 1999).

In soybean, the circadian regulatory element *circadian* is ubiquitously present in the promoter regions close to three *GmChlH*s; on the other hand, it is either absent in the 1,500 bp promoter regions of *GmChlIs* and *GmChlDs*, or present in the upstream regions far away from the genes (Table 1). These results indicate that the expression patterns of Mg-chelatase genes in soybean are similar to those in other species.

Another major type of elements in the promoters of *GmChl* genes is the hormone responsive element (HRE). It is well-known that phytohormones play important roles in chlorophyll biosynthesis pathway. For example, the greening process of seedlings is orchestrated through a complex network of interactions between auxin, gibberellic acids (GA), cytokinins (CK), ethylene, and light signal transduction pathways (Liu et al., 2017). Here, we can find auxin responsive elements, GA responsive elements, or both of them in *GmChl* promoters except for *pGmChlD2* (the promoter of *GmChlD2*), suggesting that the expression of most of *GmChl* genes can be directly regulated by the components in auxin or/and GA signaling pathway. By contrast, no CK response element is detected in any of the promoters and ethylene responsive element is only observed in *pGmChlI2a*, suggesting CK and ethylene probably indirectly regulate the transcription of *GmChlI*s, G*mChlD*s, and G*mChlH*s via interacting with other signal transduction pathways. Previous report showed that the cytokinin-mediated *Arabidopsis* root greening is dependent on transcription factor HY5 (Kobayashi et al., 2012). ETHYLENE INSENSITIVE 3 (EIN3) can active PIFs to regulate the expression of Mg-chelatase genes (Zhong et al., 2014).

Comparatively, abscisic acid (ABA) mainly functions as an inhibitor of chlorophyll biosynthesis. ABSCISIC ACID INSENSITIVE 5 (ABI5) in *Arabidopsis* represses the cotyledon greening during seed germination under light through ABA-mediated pathway (Guan et al., 2014). Defect in transcription factor ABSCISIC ACID INSENSITIVE 3 (ABI3) leads *Arabidopsis* plants producing stay-green embryos (Delmas et al., 2013). Consistently, ABA response element (ABRE) is present in most of *GmChl* promoters, except for *pGmChlI1b* and *pGmChlI2b*, showing that chlorophyll synthesis is highly controlled by ABA in soybean.

Salicylic acid (SA) and jasmonates (JA) are important signal molecules in the regulation of plant response to biotic and abiotic stress conditions, such as pathogen attacks, extreme temperatures, salts, and oxidative conditions (Khan et al., 2010; Ahmad et al., 2012; Wasternack and Hause, 2013). Chlorophyll contents usually are reduced under stress conditions (Ramachandra Reddy et al., 2004). The elements responsive to SA, including TCA-element motifs and TC-rich repeats, are relatively abundant in *pGmChlI*s and *pGmChlH*s, but rare in *pGmChlD*s, while JA response element is only found in *pGmChlI1b* and *pGmChlD2* (Table 2). It indicates that the regulations of soybean Mg-chelatase genes are different in responsive to SA and JA signals. In addition, several abiotic stress responsive elements are found in the promoters of *GmChl*s, including HSE, LTR, and MBS motifs, which are responsive to heat, low temperature, and drought, respectively (Table 2). HSE and MBS are relatively common in the promoters of 9 *GmChl* genes, and LTR only presents in *pGmChlD1* and *pGmChlHs* with a low copy number, implying the expression of *GmChls* is likely more sensitive to heat and drought.

Moreover, there are some tissue specific *cis*-elements present in the promoters of all *GmChl* genes except *GmChlI2a* (Table 2). Interestingly, GCN and skn-1 motifs related to endosperm specific expression are common and abundant in *GmChl* genes, indicating Mg-chelatase in endosperm are likely functionally important. Additionally, two copies of CAT-box motifs related to root meristem development are found in *pGmChlI1a* and *pGmChlD1*, and one copy of HD-Zip1 related to leaf development is found in *GmChlD2* (Table 2).

Collectively, regulatory *cis*-elements response to environmental and growth conditions are diverse among the promoters of different Mg-chelatase genes, indicating that the expression of soybean Mg-chelatase genes are complicated and differentially regulated by various factors.

### Subcellular localization of GmChlIs, GmChlDs, and GmChlHs

**Figure 3:**
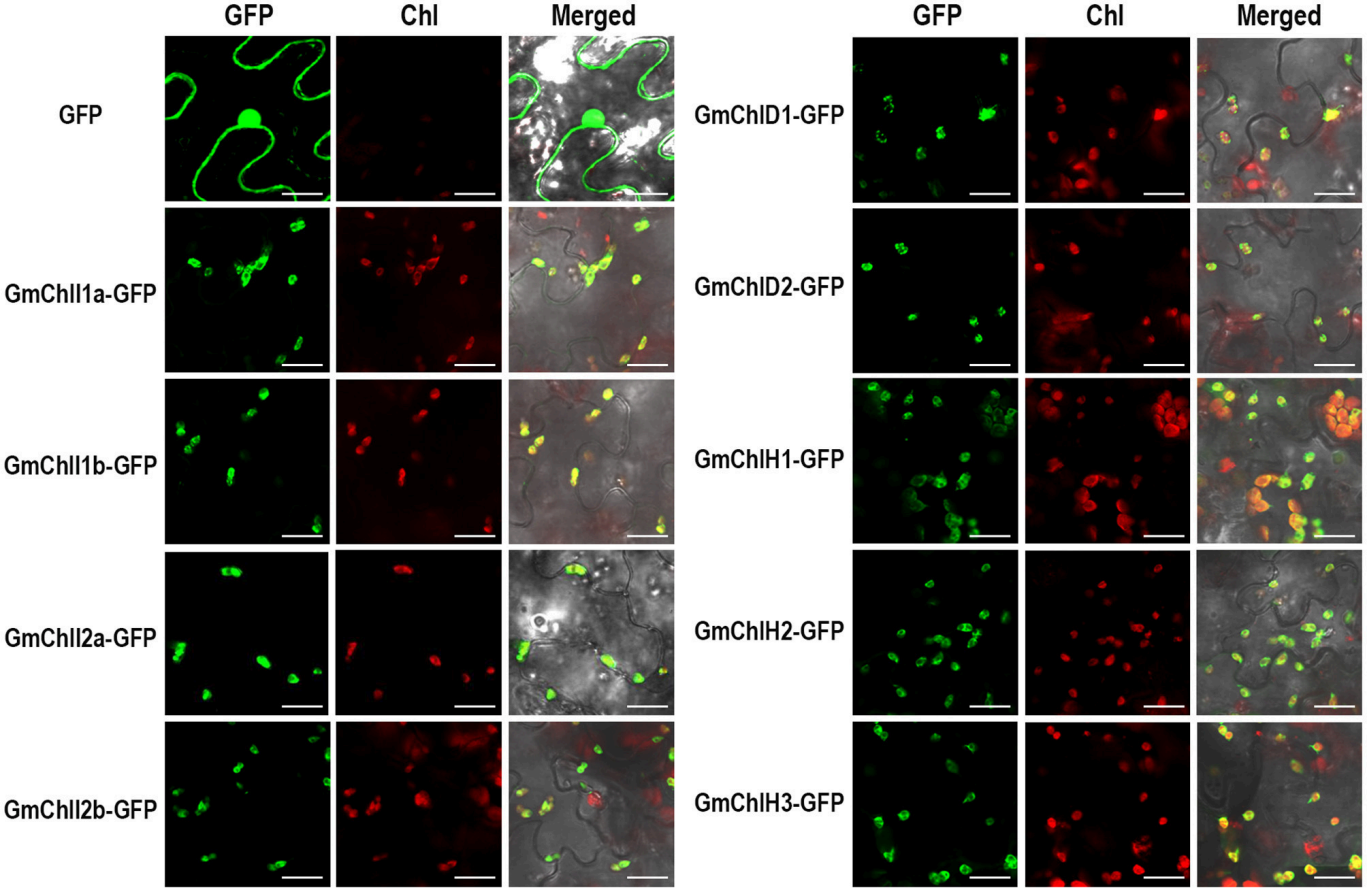
Subcellular localization of GmChlIs, GmChlDs, and GmChlHs. Free GFP and GmChlIs, GmChlDs, and GmChlHs tagged with a C-terminal GFP were transiently expressed under the control of 35S promoter in *N. benthamina* leaves and observed by confocal microscopy. In each case, images of GFP fluorescence (GFP), chlorophyll autofluorescence (Chl), and merged GFP and chlorophyll fluorescence with bright-field (Merged) are shown. Scale bar = 20 μm.

Sequence alignment analysis showed that GmChlIs, GmChlDs, and GmChlHs all contain potential CTP at the N-terminus similar to AtChlI1, AtChlD, and AtChlH (Figures S1–S3). To confirm their subcellular localizations, we generated GFP fusion proteins by fusing *GFP* to the C termini of full-length *GmChlI*s, *GmChlD*s, and *GmChlH*s, respectively. Nine fusion proteins were transiently expressed in the leaf tissue of *N. benthamiana* through agro-infiltration, and observed under confocal microscope 2 days after inoculation. The results clearly demonstrated that GFP-fused GmChlI, GmChlD, and GmChlH subunits were expressed and localized in the chloroplasts, as shown by the co-localization of GFP signal and chlorophyll autofluorescence (Figure 3), supporting that GmChlIs, GmChlDs, and GmChlHs are chloroplastic proteins, in agreement with their expected functions.

### Interactions among GmChlIs, GmChlDs, and GmChlHs

It has been established that ChlI, ChlD, and ChlH are assembled together to form an active Mg-chelatase holo-complex (Adhikari et al., 2011). We performed Y2H and BiFC assay to test these interactions among GmChlIs, GmChlDs, and GmChlHs.

**Figure 4:**
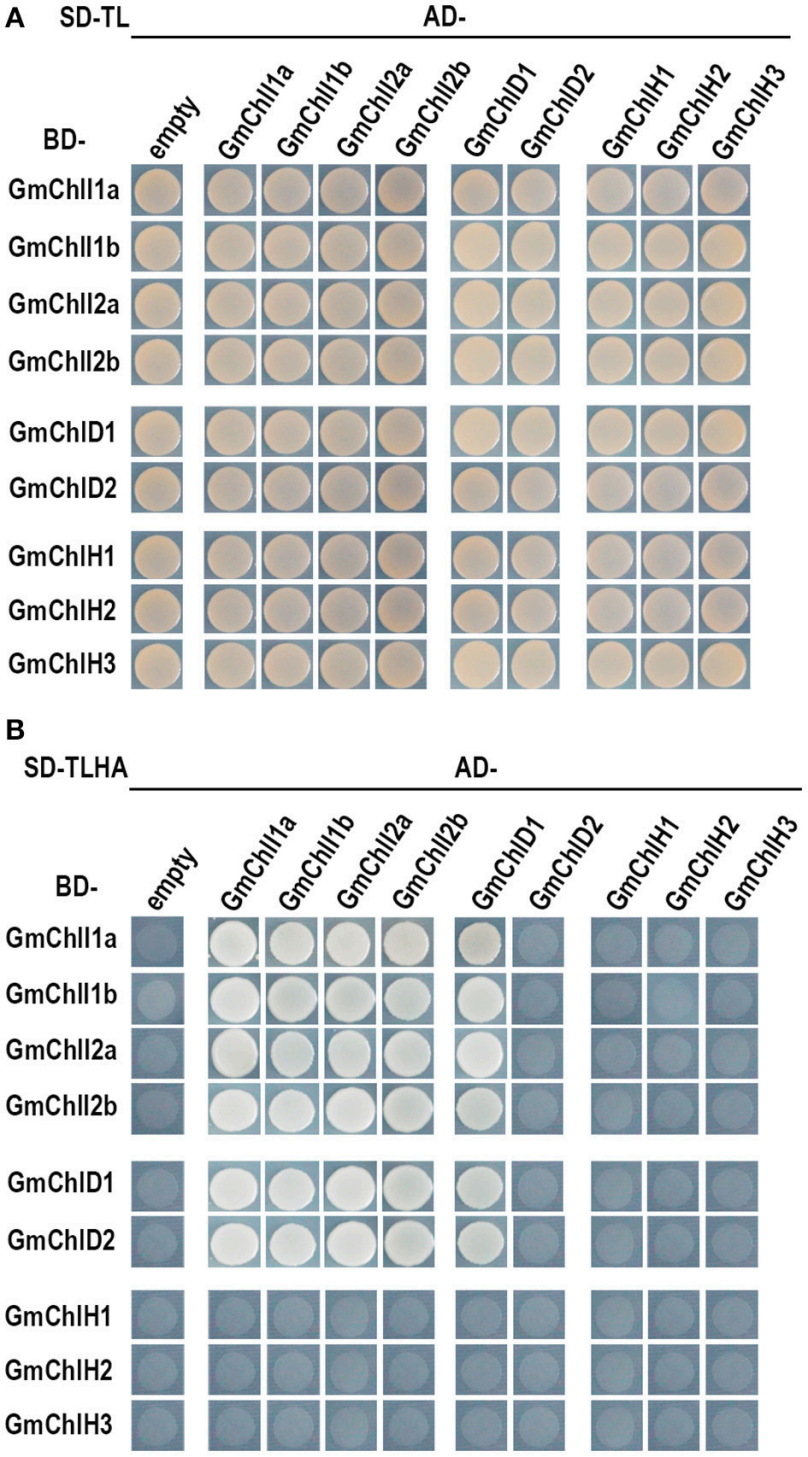
Yeast two hybrid assay between GmChlIs, GmChlDs, and GmChlHs. pGBKT7 vectors expressing BD-fused GmChlIs, GmChlDs, and GmChlHs were co-transformed with pGADT7 empty vector or the ones expressing AD-fused GmChlIs, GmChlDs, and GmChlHs into the yeast strain AH109. The transformants were grown **(A)** on the synthetic dextrose medium (SD) lack of Trp and Leu (SD-TL) or **(B)** on the SD medium missing Trp, Leu, His, and Ade (SD-TLHA).

The Y2H assays revealed that each GmChlI isoform can interact with itself and the other paralogs (Figure 4), suggesting that four GmChlIs are able to form homo- and hetero- hexameric ring. Similarly, they also interact with GmChlD1, indicating that they can form the oligomer with GmChlD1 protein. Meanwhile, GmChlD1 could interact with itself, which suggesting GmChlD1 can form the hexameric ring like GmChlIs. Notably, when GmChlD2 was fused with Gal4 activation domain, it did not interact with GmChlI or GmChlD proteins; but the interactions between them could be observed when GmChlD2 was fused with Gal4 DNA-binding domain (Figure 4), indicating that Gal4 activation domain could interfere the interaction of GmChlD2. In addition, no interaction was detected between a GmChlH isoform and any soybean Mg-chelatase subunit through Y2H method (Figure 4).

During BiFC experiments, obvious reconstructed YFP fluorescence was observed in chloroplasts coexpressing GmChlIs-YFP_N_ with GmChlIs-YFPc, GmChlDs-YFPc, or GmChlHs-YFPc (Figure S4). It confirmed the interactions between GmChlIs and GmChlDs detected in Y2H, and suggested that 4 GmChlIs could interact with all the GmChlH isoforms. Similar results were obtained by coexpression of GmChlDs-YFP_N_ with GmChlIs-YFPc, GmChlDs-YFPc, or GmChlHs-YFPc, verifying GmChlDs could interact with GmChlIs and GmChlDs, and implying the interactions between GmChlDs and GmChlHs as well (Figure S5). In addition, the interactions between GmChlHs and GmChlIs or GmChlDs were observed when GmChlHs-YFP_N_ was co-expressed with GmChlIs-YFPc or GmChlDs-YFPc in leaves (Figure S6). The interactions between GmChlH and other subunits were not observed in Y2H experiment possibly because GmChlH protein can only interact with GmChlI or GmChlD proteins in the two-tiered ring complex but not to the single GmChlI or GmChlD hexameric ring. As the negative control, no YFP signal was detected in the leaves co-transformed with a GmChlI-YFP_N_, GmChlD-YFP_N_, or GmChlH-YFP_N_ construct in combination with an empty YFP_C_ vector. Taken together, these results suggest that all of GmChlIs, GmChlDs, and GmChlHs are likely involved in Mg-chelatase activity by physically interacting with each other.

### Ecotopic expression of *GmChlIs, GmChlDs*, and *GmChlHs* in *Arabidopsis* corresponding mutants

**Figure 5:**
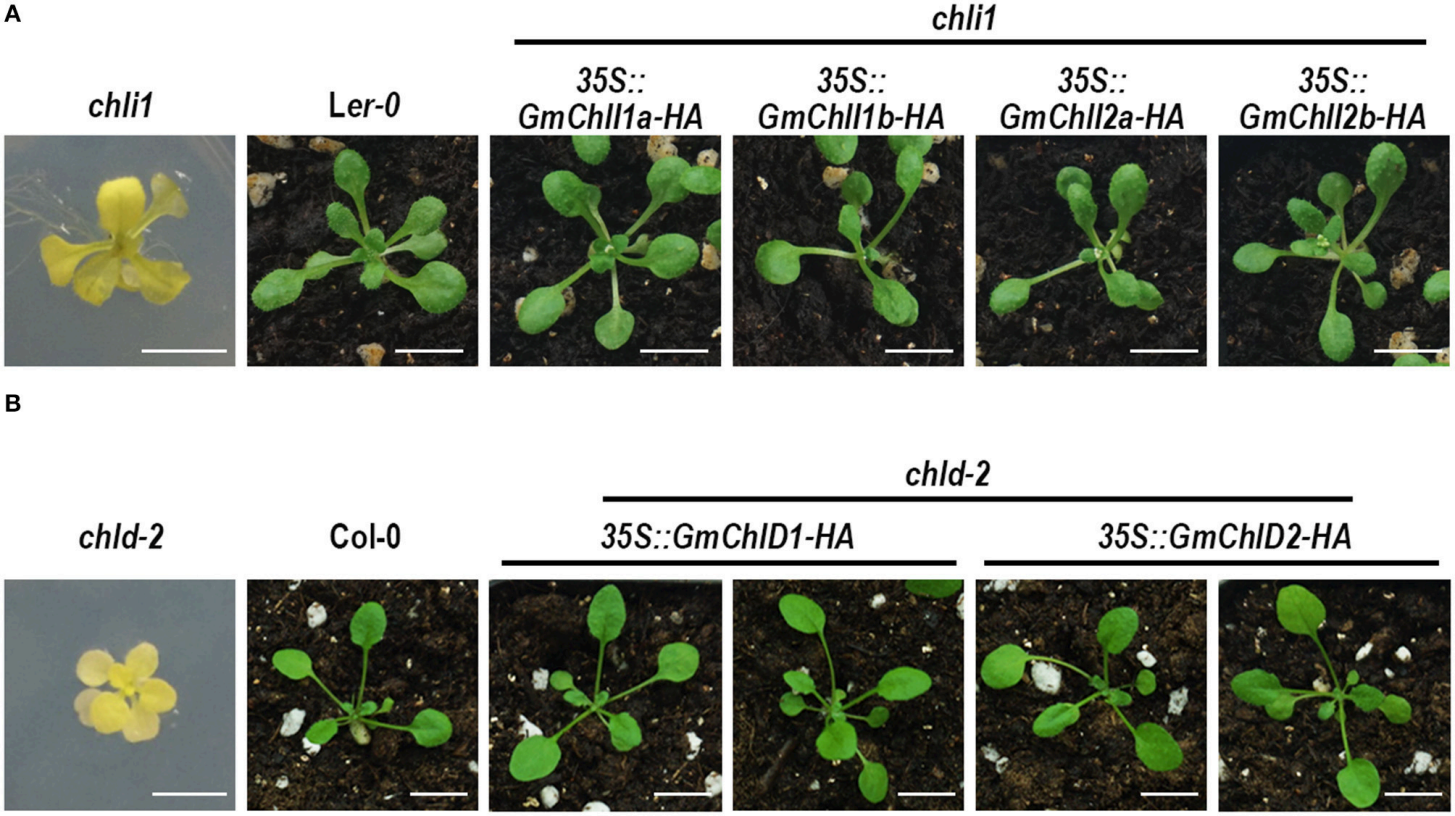
Phenotypes of *Arabidopsis chli1* and *chld-2* mutants carrying *GmChlI* and *GmChlD* genes**. (A)** 3-week old plants of *chli1* mutant (L*er-0* background), L*er-0*, and transgenic *chli1* mutants carrying 35S-driven C-terminal *HA* fused *GmChlI1a, GmChlI1b, GmChlI2a*, or*GmChlI2b*; **(B)** 3-week old plants of *chld-2* mutant (Col-0 background), Col-0, and transgenic *chld-2* mutants carrying 35S-driven C-terminal *HA* fused *GmChlD1* or *GmChlD2*. All transgenic genes fully complement the mutant phenotypes. Scale bars = 5 mm.

Evidences above imply that GmChlIs, GmChlDs, and GmChlHs are likely functional Mg-chelatase subunits. For further verification, we performed a complementation test by ectopically expressing *GmChlI, GmChlD*, and *GmChlH* genes in *Arabidopsis* corresponding mutants, *chli1, chld-2*, and *gun5-2. chli1* and *chld-2* are T-DNA insertional knockout and knockdown mutants respectively, Figure S7), showing seedling-lethal phenotypes (Figure 5). Therefore, all 3*5S::GmChlI-HA* and *35S::GmChlD-HA* constructs were transformed into corresponding heterozygous mutants, and the transgenic plants in homozygous mutant background were identified by PCR afterwards. At the T_3_ generation, we found that homozygous *chli1* mutants carrying *35S::GmChlI1a-HA, GmChlI1b-HA, GmChlI2a-HA*, or *GmChlI2b-HA* showed wild-type green phenotype (Figure 5A). Similar wild-type phenotype was observed in homozygous *chld-2* mutants carrying *35S::GmChlD1-HA* or *GmChlD2-HA* (Figure 5B). These results demonstrated that all GmChlI and GmChlD isoforms could play a full functional role in the Mg-chelatase holoenzyme.

**Figure 6:**
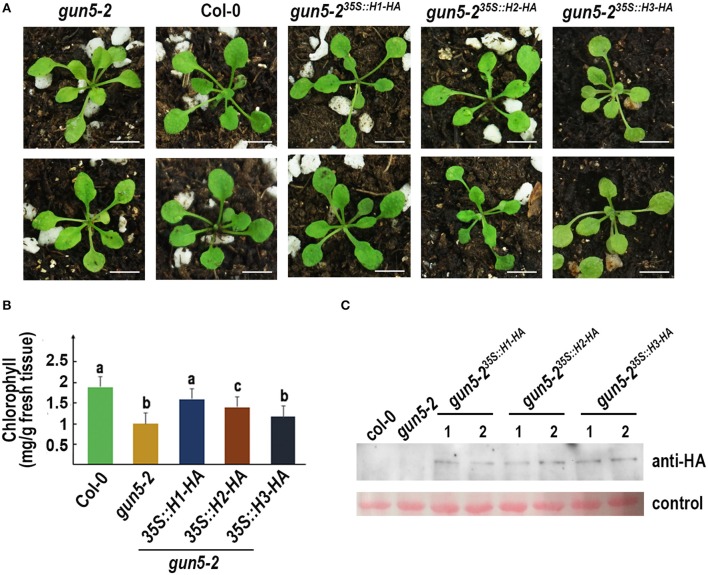
Phenotypes of *Arabidopsis gun5-2* mutants carrying *GmChlH* genes. **(A)** 3-week old plants of *gun5-2* mutant (Col-0 background), Col-0, and transgenic *gun5-2* mutants carrying 35S-driven C-terminal *HA* fused *GmChlH1, GmChlH2*, and *GmChlH3*. Two independent transgenic lines are shown for each gene; **(B)** Chlorophyll contents in 3-week old plants from the lines present in **(A)**. Error bars indicate sd from three biological replications. Student *t*-test was performed for statistical analysis among all the lines; different letters represent statistically significant differences between transgenic plant and wild-type or mutant lines (*P* < 0.05); **(C)** Protein expression level of transgenes in the lines present in **(A)**. Western blot analysis was performed using HA tag antibody. The Ponceau S staining of Rubisco large subunit is used as the loading control. Scale bars = 5 mm.

*Arabidopsis gun5-2* mutant, an *AtChlH* knock-down mutant, harbors a T-DNA insertion at the promoter region of *AtChlH* (Figure S7) and produces viable pale green seedlings (Figure 6A). The 35S promoter-driven *HA* fused *GmChlH1, GmChlH2*, and *GmChlH3* were transformed into the homozygous *gun5-2* mutant. Transgenic plants carrying *35S::GmChlH1-HA* look as green as wild-type plants; however, transgenic plants carrying *35S::GmChlH2-HA* and *35S::GmChlH3-HA* show intermediate pale-green phenotypes (Figure 6A).

Results from a detailed chlorophyll analysis were consistent to the phenotypes (Figure 6B). The *gun5-2* plants expressing different GmChlHs had different chlorophyll levels. The chlorophyll content in *gun5-2*^35*S*::*GmChlH*1−*HA*^ plants was highest, similar to that in wild types, and *gun5-2*^35*S*::*GmChlH*3−*HA*^ plants had lowest amount of chlorophyll, which seemed a little higher than that in *gun5-2*, but without statistically significant difference (Figure 6B). As to *gun5-2* ^35*S*::*GmChlH*2−*HA*^ plants, the chlorophyll content was lower than that in *gun5-2*^35*S*::*GmChlH*1−*HA*^ plants but higher than that in *gun5-2*^35*S*::*GmChlH*3−*HA*^ plants (Figure 6B). However, western blot analysis exhibited that protein contents of three GmChlHs were similar in corresponding transgenic plants (Figure 6C), indicating that the failure of GmChlH2-HA and GmChlH3-HA to fully complement the *gun5-2* mutant phenotypes was not caused by a lack or lower of gene expression. These results imply that GmChlH1 is probably a full functional ChlH subunit, whereas GmChlH2 and GmChlH3 have lower chelatase activities.

## Conclusion

In cyanobacterium and monocotyledon plants, each subunit of Mg-chelatase has only one isoform (Jensen et al., 1996; Sawers et al., 2006; Zhang et al., 2006; Muller et al., 2014), whereas most dicotyledons encodes 2 ChlI, 1 ChlD, and 1 ChlH subunits (Du et al., 2012; Figure 1). Soybean genome harbors 4 *GmChlI*, 2 *GmChlD*, and 3 *GmChlH* genes, and other legume species have 2 *ChlI*, 1 *ChlD*, and 2 *ChlH* genes (Figures 1, 2). Phylogenetics analysis reveals that there are two gene duplication events occurring in the history of soybean Mg-chelatase, including one at the origin of the legume family, and the other one after speciation of soybean (Figure 2). The first round of gene duplication seems only apply to *GmChlI* and *GmChlH* genes. This is in an agreement with two recent major duplication events in soybean genome at 58 and 13 million years ago revealed by the soybean genome sequencing project (Schmutz et al., 2010).

Sequence alignment analysis (Figures S1–S3) and interaction assay (Figure 4; Figures S4–S6) indicate that the isoforms of each soybean Mg-chelatase subunit are likely functional proteins. Further ectopic expression of each Mg-chelatase subunit in *Arabidopsis* confirms that all of GmChlIs and GmChlDs have proper biochemical function because they can fully recover chlorophyll biosynthesis of the corresponding *Arabidopsis* mutants (Figure 5). However, the experiment also reveals that GmChlH2 and GmChlH3 are not as active as GmChlH1 in *Arabidopsis* (Figure 6). Even though GmChlH1, GmChlH2, and GmChlH3 are very similar in primary structure, the fine difference in their sequences could lead to a big change in enzyme activity.

Results from qRT-PCR and in silicon promoter analyses indicate that all soybean Mg-chelatase subunits are highly and similarly regulated by light, concerning the tissue specific expression patterns and the number of light response elements (Tables 1, 2; Figure 2). Nevertheless, the expression levels are varied among different isoforms (Figure 2). Especially, the transcriptions of *GmChlI2a* and *GmChlI2b* are extremely low compared to *GmChlI1a* and *GmChlI1b* in photosynthetic tissues, indicating the latter two play the major role during active photosynthesis. Moreover, the elements responsive to environmental and growth conditions other than LREs are diverse in the promoters of all the subunit genes, indicating they are differentially regulated.

With respect to the sequence, biochemical function, and gene expression and regulation, we conclude that the paralogs of each soybean Mg-chelatase subunit are diverged in biological functions. The differently expressed and variously functional isoforms of the Mg-chelatase subunits may also suggest that there is a more complex regulatory mechanism to control chlorophyll content in soybean in order to response to different development stages and environmental stresses, and to optimize its light harvesting capacity and photosynthetic efficiency.

## Author contributions

MX, AF, and DZ designed research. DZ, EC, XY, YoC, QY, YaC, and XL performed research. DZ, EC, XY, YoC, QY, YW, MX, and AF analyzed data. DZ, MX, and AF wrote the paper with contributions from all authors. All authors read and approved the manuscript.

### Conflict of interest statement

The authors declare that the research was conducted in the absence of any commercial or financial relationships that could be construed as a potential conflict of interest.

## References

[B1] Adhikari N. D. Froehlich J. E. Strand D. D. Buck S. M. Kramer D. M. Larkin R. M. (2011). GUN4-porphyrin complexes bind the ChlH/GUN5 subunit of Mg-chelatase and promote chlorophyll biosynthesis in *Arabidopsis*. Plant Cell 23, 1449–1467. 10.1105/tpc.110.082503 21467578 PMC3101535

[B2] Ahmad I. Khaliq T. Ahmad A. Basra S. M. A. Hasnain Z. Ali A. (2012). Effect of seed priming with ascorbic acid, salicylic acid and hydrogen peroxide on emergence, vigor and antioxidant activities of maize. Afr. J. Biotechnol. 11, 1127–1132. 10.5897/AJB11.2266

[B3] Ainsworth E. A. Yendrek C. R. Skoneczka J. A. Long S. P. (2012). Accelerating yield potential in soybean: potential targets for biotechnological improvement. Plant Cell Environ. 35, 38–52. 10.1111/j.1365-3040.2011.02378.x 21689112

[B4] Bi W. Wu L. Coustry F. de Crombrugghe B. Maity S. N. (1997). DNA binding specificity of the CCAAT-binding factor CBF/NF-Y. J. Biol. Chem. 272, 26562–26572. 10.1074/jbc.272.42.26562 9334236

[B5] Brzezowski P. Richter A. S. Grimm B. (2015). Regulation and function of tetrapyrrole biosynthesis in plants and algae. Biochim. Biophys. Acta. 1847, 968–985. 10.1016/j.bbabio.2015.05.007 25979235

[B6] Campbell B. W. Mani D. Curtin S. J. Slattery R. A. Michno J. M. Ort D. R.. (2015). Identical substitutions in magnesium chelatase paralogs result in chlorophyll-deficient soybean mutants. G3. (Bethesda) 5, 123–131. 10.1534/g3.114.015255 25452420 PMC4291463

[B7] Chen M. Blankenship R. E. (2011). Expanding the solar spectrum used by photosynthesis. Trends Plant Sci. 16, 427–431. 10.1016/j.tplants.2011.03.011 21493120

[B8] Chen X. Pu H. Fang Y. Wang X. Zhao S. Lin Y.. (2015). Crystal structure of the catalytic subunit of magnesium chelatase. Nat. Plants 1:15125. 10.1038/nplants.2015.125 27250678

[B9] Clough S. J. Bent A. F. (1998). Floral dip: a simplified method for *Agrobacterium*-mediated transformation of *Arabidopsis thaliana*. Plant J. 16, 735–743. 10.1046/j.1365-313x.1998.00343.x 10069079

[B10] Croce R. van Amerongen H. (2014). Natural strategies for photosynthetic light harvesting. Nat. Chem. Biol. 10, 492–501. 10.1038/nchembio.1555 24937067

[B11] Delmas F. Sankaranarayanan S. Deb S. Widdup E. Bournonvile C. Bollier N.. (2013). ABI3 controls embryo degreening through Mendel's I locus. Proc. Natl. Acad. Sci. U.S.A. 110, E3888–E3894. 10.1073/pnas.1308114110 24043799 PMC3791760

[B12] Deng X. J. Zhang H. Q. Wang Y. He F. Liu J. L. Xiao X.. (2014). Mapped clone and functional analysis of leaf-color gene *Ygl7* in a rice hybrid (*Oryza sativa* L. ssp. indica). PLoS ONE 9:e99564. 10.1371/journal.pone.0099564 24932524 PMC4059691

[B13] Dornbos D. L. Jr. Mullen R. E. (1992). Soybean seed protein and oil contents and fatty acid composition adjustments by drought and temperature. J. Am. Oil Chem. Soc. 69, 228–231. 10.1007/BF02635891

[B14] Du S. Y. Zhang X. F. Lu Z. Xin Q. Wu Z. Jiang T.. (2012). Roles of the different components of magnesium chelatase in abscisic acid signal transduction. Plant Mol. Biol. 80, 519–537. 10.1007/s11103-012-9965-3 23011401 PMC3472068

[B15] Egli D. B. (2008). Soybean yield trends from 1972 to 2003 in mid-western USA. Field Crops Res. 106, 53–59. 10.1016/j.fcr.2007.10.014

[B16] Fodje M. N. Hansson A. Hansson M. Olsen J. G. Gough S. Willows R. D.. (2001). Interplay between an AAA module and an integrin I domain may regulate the function of magnesium chelatase. J. Mol. Biol. 311, 111–122. 10.1006/jmbi.2001.4834 11469861

[B17] Gibson L. C. Marrison J. L. Leech R. M. Jensen P. E. Bassham D. C. Gibson M.. (1996). A putative Mg chelatase subunit from *Arabidopsis thaliana* cv C24 (sequence and transcript analysis of the gene, import of the protein into chloroplasts, and *in situ* localization of the transcript and protein). Plant Physiol. 111, 61–71. 10.1104/pp.111.1.61 8685276 PMC157813

[B18] Gräfe S. Saluz H. P. Grimm B. Hänel F. (1999). Mg-chelatase of tobacco: the role of the subunit CHLD in the chelation step of protoporphyrin IX. Proc. Natl. Acad. Sci. U.S.A. 96, 1941–1946. 10.1073/pnas.96.5.1941 10051574 PMC26716

[B19] Guan C. Wang X. Feng J. Hong S. Liang Y. Ren B.. (2014). Cytokinin antagonizes abscisic acid-mediated inhibition of cotyledon greening by promoting the degradation of ABSCISIC ACID INSENSITIVE5 protein in *Arabidopsis*. Plant Physiol. 164, 1515–1526. 10.1104/pp.113.234740 24443524 PMC3938637

[B20] Hansson A. Willows R. D. Roberts T. H. Hansson M. (2002). Three semidominant barley mutants with single amino acid substitutions in the smallest magnesium chelatase subunit form defective AAA+ hexamers. Proc. Natl. Acad. Sci. U.S.A. 99, 13944–13949. 10.1073/pnas.212504499 12357035 PMC129802

[B21] Huang Y. S. Li H. M. (2009). *Arabidopsis* CHLI2 can substitute for CHLI1. Plant Physiol. 50, 636–645. 10.1104/pp.109.135368 PMC2689973 19363094

[B22] Jensen P. E. Gibson L. C. Hunter C. N. (1998). Determinants of catalytic activity with the use of purified I, D and H subunits of the magnesium protoporphyrin IX chelatase from *Synechocystis* PCC6803. Biochem. J. 334, 335–344. 10.1042/bj3340335 9716491 PMC1219695

[B23] Jensen P. E. Gibson L. C. Henningsen K. W. Hunter C. N. (1996). Expression of the chlI, chlD, and chlH genes from the *Cyanobacterium synechocystis* PCC6803 in *Escherichia coli* and demonstration that the three cognate proteins are required for magnesium-protoporphyrin chelatase activity. J. Biol. Chem. 271, 16662–16667. 10.1074/jbc.271.28.16662 8663186

[B24] Karger G. A. Reid J. D. Hunter C. N. (2001). Characterization of the binding of deuteroporphyrin IX to the magnesium chelatase H subunit and spectroscopic properties of the complex. Biochemistry 40, 9291–9299. 10.1021/bi010562a 11478896

[B25] Khan N. A. Syeed S. Masood A. Nazar R. Iqbal N. (2010). Application of salicylic acid increases contents of nutrients and antioxidative metabolism in mungbean and alleviates adverse effects of salinity stress. Int. J. Plant Biol. 1:e1 10.4081/pb.2010.e1

[B26] Kim S. Schlicke H. Van R. K. Karvonen K. Subramaniam A. Richter A. S.. (2013). *Arabidopsis* chlorophyll biosynthesis: an essential balance between the methylerythritol phosphate and tetrapyrrole pathways. Plant Cell 25, 4984–4993. 10.1105/tpc.113.119172 24363312 PMC3904000

[B27] Kobayashi K. Baba S. Obayashi T. Obayashi T. Sato M. Toyooka K.. (2012). Regulation of root greening by light and auxin/cytokinin signaling in *Arabidopsis*. Plant Cell 24, 1081–1095. 10.1105/tpc.111.092254 22415275 PMC3336121

[B28] Koester R. P. Skoneczka J. A. Cary T. R. Diers B. W. Ainsworth E. A. (2014). Historical gains in soybean (*Glycine max* Merr.) seed yield are driven by linear increases in light interception, energy conversion, and partitioning efficiencies. J. Exp. Bot. 65, 3311–3321. 10.1093/jxb/eru187 24790116 PMC4071847

[B29] Lake V. Olsson U. Willows R. D. Hansson M. (2004). ATPase activity of magnesium chelatase subunit I is required to maintain subunit D *in vivo*. Eur. J. Biochem. 271, 2182–2188. 10.1111/j.1432-1033.2004.04143.x 15153108

[B30] Lescot M. Déhais P. Thijs G. Marchal K. Moreau Y. Peer Y. V. D.. (2002). PlantCARE, a database of plant *cis*-acting regulatory elements and a portal to tools for in silico analysis of promoter sequences. Nucleic Acids Res. 30, 325–327. 10.1093/nar/30.1.325 11752327 PMC99092

[B31] Liu X. Li Y. Zhong S. (2017). Interplay between light and plant hormones in the control of *Arabidopsis* seedling chlorophyll biosynthesis. Front. Plant Sci. 8:1433. 10.3389/fpls.2017.01433 28861105 PMC5562715

[B32] Masuda T. (2008). Recent overview of the Mg branch of the tetrapyrrole biosynthesis leading to chlorophylls. Photosynth. Res. 96, 121–143. 10.1007/s11120-008-9291-4 18273690

[B33] Masuda T. Fujita Y. (2008). Regulation and evolution of chlorophyll metabolism. Photochem. Photobiol. Sci. 7, 1131–1149. 10.1039/b807210h 18846277

[B34] Masuda T. Goldsmith P. D. (2009). World soybean production: area harvested, yield, and long-term projections. Int. Food Agribus. Manag. Rev. 12, 143–161.

[B35] Mochizuki N. Brusslan J. A. Larkin R. Nagatani A. Chory J. (2001). *Arabidopsis genomes uncoupled 5* (*GUN5*) mutant reveals the involvement of Mg-chelatase H subunit in plastid-to-nucleus signal transduction. Proc. Natl. Acad. Sci. U.S.A. 98, 2053–2058. 10.1073/pnas.98.4.2053 11172074 PMC29380

[B36] Müller A. H. Sawicki A. Zhou S. Tabrizi S. T. Luo M. Hansson M.. (2014). Inducing the oxidative stress response in *Escherichia coli* improves the quality of a recombinant protein: magnesium chelatase ChlH. Protein Exp. Purif. 101, 61–67. 10.1016/j.pep.2014.06.004 24931499

[B37] Nakayama M. Masuda T. Bando T. Yamagata H. Ohta H. Takamiya K. (1998). Cloning and expression of the soybean chlH gene encoding a subunit of Mg-chelatase and localization of the Mg^2+^ concentration-dependent ChlH protein within the chloroplast. Plant Cell Physiol. 39, 275–284. 10.1093/oxfordjournals.pcp.a029368 9588025

[B38] Nakayama M. Masuda T. Sato N. Yamagata H. Bowler C. Ohta H. (1995). Cloning, subcellular localization and expression of *chlI*, a subunit of magnesium chelatase in soybean. Biochem. Biophys. Res. Commun. 215, 422–428. 10.1006/bbrc.1995.2481 7575622

[B39] Natarajan S. Luthria D. Bae H. Lakshman D. Mitra A. (2013). Transgenic soybeans and soybean protein analysis: an overview. J. Agric. Food Chem. 61, 1736–1743. 10.1021/jf402148e 24099420

[B40] Palmer R. G. Hedges B. R. Benavente R. S. Groose R. W. (1989). *w4*-mutable line in soybean. Dev. Genet. 10, 542–551. 10.1002/dvg.1020100613

[B41] Papenbrock J. Mock H. Kruse E. Grimm B. (1999). Expression studies in tetrapyrrole biosynthesis: inverse maxima of magnesium chelatase and ferrochelatase activity during cyclic photoperiods. Planta 208, 264–273. 10.1007/s004250050558

[B42] Pettigrew W. T. Hesketh D. Peters D. B. Woolley T. (1989). Characterization of canopy photosynthesis of chlorophyll-deficient soybean isolines. Crop Sci. 29, 1024–1028. 10.2135/cropsci1989.0011183X002900040040x

[B43] Ramachandra Reddy A. Chaitanya K. V. Vivekanandan M. (2004). Drought-induced responses of photosynthesis and antioxidant metabolism in higher plants. J. Plant Physiol. 161, 1189–1202. 10.1016/j.jplph.2004.01.013 15602811

[B44] Sawers R. J. Viney J. Farmer P. R. Bussey R. R. Olsefski G. Anufrikova K.. (2006). The maize *Oil yellow1* (*Oy1*) gene encodes the I subunit of magnesium chelatase. Plant Mol. Biol. 60, 95–106. 10.1007/s11103-005-2880-0 16463102

[B45] Schlueter J. A. Lin J. Y. Schlueter S. D. VasylenkoSanders I. F. Deshpande S. Yi J.. (2007). Gene duplication and paleopolyploidy in soybean and the implications for whole genome sequencing. BMC Genomics 8:330. 10.1186/1471-2164-8-330 17880721 PMC2077340

[B46] Schmutz J. Cannon S. B. Schlueter J. Ma J. Mitros T. Nelson W.. (2010). Genome sequence of the palaeopolyploid soybean. Nature 463, 178–183. 10.1038/nature08670 20075913

[B47] Shoemaker R. C. Schlueter J. Doyle J. J. (2006). Paleopolyploidy and gene duplication in soybean and other legumes. Curr. Opin. Plant Biol. 9, 104–109. 10.1016/j.pbi.2006.01.007 16458041

[B48] Sirijovski N. Olsson U. Lundqvist J. Al-Karadaghi S. Willows R. D. Hansson M. (2006). ATPase activity associated with the magnesium chelatase H-subunit of the chlorophyll biosynthetic pathway is an artefact. Biochem. J. 400, 477–484. 10.1042/BJ20061103 16928192 PMC1698598

[B49] Slattery R. A. VanLoocke A. Bernacchi C. J. Zhu X. G. Ort D. R. (2017). Photosynthesis, light use efficiency, and yield of reduced-chlorophyll soybean mutants in field conditions. Front. Plant Sci. 8:549. 10.3389/fpls.2017.00549 28458677 PMC5394119

[B50] Stephenson P. G. Terry M. J. (2008). Light signaling pathways regulating the Mg-chelatase branchpoint of chlorophyll synthesis during de-etiolation in *Arabidopsis thaliana*. Photochem. Photobiol. Sci. 7, 1243–1252. 10.1039/b802596g 18846290

[B51] Tamura K. Stecher G. Peterson D. Filipski A. Kumar S. (2013). MEGA6: Molecular evolutionary genetics analysis version 6.0. Mol. Biol. Evol. 30, 2725–2729. 10.1093/molbev/mst197 24132122 PMC3840312

[B52] Toledo-Ortiz G. Johansson H. Lee K. P. Bou-Torrent J. Stewart K. Steel G.. (2014). The HY5-PIF regulatory module coordinates light and temperature control of photosynthetic gene transcription. PLoS Genet. 10:e1004416. 10.1371/journal.pgen.1004416 24922306 PMC4055456

[B53] Waadt R. Schmidt L. K. Lohse M. Hashimoto K. Bock R. Kudla J. (2008). Multicolor bimolecular fluorescence complementation reveals simultaneous formation of alternative CBL/CIPK complexes in planta. Plant J. 56, 505–516. 10.1111/j.1365-313X.2008.03612.x 18643980

[B54] Walker B. J. Drewry D. T. Slattery R. A. VanLoocke A. Cho Y. B. Ort D. R. (2017). Chlorophyll can be reduced in crop canopies with little penalty to photosynthesis. Plant Physiol. 176, 1215–1232. 10.1104/pp.17.01401 29061904 PMC5813550

[B55] Walker C. J. Willows R. D. (1997). Mechanism and regulation of Mg-chelatase. Biochem. J. 327, 321–333. 10.1042/bj3270321 9359397 PMC1218797

[B56] Wasternack C. Hause B. (2013). Jasmonates: biosynthesis, perception, signal transduction and action in plant stress response, growth and development. Ann. Bot. 111, 1021–1058. 10.1093/aob/mct067 23558912 PMC3662512

[B57] Winter D. Vinegar B. Nahal H. Ammar R. Wilson G. V. Provart N. (2007). An “electronic fluorescent pictograph” browser for exploring and analyzing large-scale biological data sets. PLoS ONE 2:e718. 10.1371/journal.pone.0000718 17684564 PMC1934936

[B58] Zhang H. Li J. Yoo J. H. Yoo S. C. Cho S. H. Koh H. J.. (2006). Rice *Chlorina-1* and *Chlorina-9* encode ChlD and ChlI subunits of Mg-chelatase, a key enzyme for chlorophyll synthesis and chloroplast development. Plant Mol. Biol. 62, 325–337. 10.1007/s11103-006-9024-z 16915519

[B59] Zhong S. Shi H. Xue C. Wei N. Guo H. Deng X. W. (2014). Ethylene-orchestrated circuitry coordinates a seedling's response to soil cover and etiolated growth. Proc. Natl. Acad. Sci. U.S.A. 111, 3913–3920. 10.1073/pnas.1402491111 24599595 PMC3964075

[B60] Zhu X. G. Long S. P. Ort D. R. (2010). Improving photosynthetic efficiency for greater yield. Annu. Rev. Plant Biol. 61, 235–261. 10.1146/annurev-arplant-042809-112206 20192734

