# Supplementary material for: Molecular Characterization of Magnesium Chelatase in Soybean [Glycine max (L.) Merr.]
Source: Front Plant Sci. 2018 Jun 19;9:720. doi: 10.3389/fpls.2018.00720 (PMC6018531; doi:10.3389/fpls.2018.00720)
Supplement: Supplementary file 1 [file Data_Sheet_1.pdf]

Supplementary Table S1. PCR primers used in this study.

Description	Primer Name	Sequence (5'→3')
**Cloning full-length cDNA**		
GmChlI1a	GmChlI1a-1 F	ATGGCGTCCGCCTTGGGCACTTCTTCA
	GmChlI1a-1 R	TCAGCTGAATACCTCATAAAATTTCTC
GmChlI1b	GmChlI1b-1 F	ATGGCGTCCACGTTGGGCACTTCTTCAA
	GmChlI1b-1 R	TCAGCTAAATACCTCATAAAATTTCTC
GmChlI2a	GmChlI2a-1 F	ATGGCGTCCATGTTTGGCACATCTTC
	GmChlI2a-1 R	TCATCTGAAAACCTCGTAAAATTTCTC
GmChlI2b	GmChlI2b-1 F	ATGGCTTCCACGTTTGGCGCATCTTC
	GmChlI2b-1 R	TCATCTGAAAACCTCATAAAATTTCTC
GmChlD1	GmChlD1-1 F	ATGGGTTTCGCTTTGGCATACACAGC
	GmChlD1-1 R	TCATGAACTTTTCAAAGCTGATAAAG
GmChlD2	GmChlD2-1 F	ATGGGTTTCGCTTTGGCATTCACAGC
	GmChlD2-1 R	TCATGAACTTTTCAAAGCTGATAAAG
GmChlH1	GmChlH1-1 F	TCCAACAACCGCTCATCATTC
	GmChlH1-1 R	ATTACCAAATTCTTTTCATTC
GmChlH2	GmChlH2-1 F	CCGAATATTTTTCAATTCAAGC
	GmChlH2-1 R	TATTCATTACCAAATTCTTTTC
GmChlH3	GmChlH3-1 F	AAAGGAGAAAAAGGAAAAAAGAG
	GmChlH3-1 R	GCGTGTCATTATGTTCAATAAG
**Real-time quantitative PCR**		
actin11	Gm_actin11-qPCR-F	CGGTGGTTCTATCTTGGCATC
	Gm_actin11-qPCR-R	GTCTTTCGCTTCAATAACCCTA
GmChlI1a	GmChlI1a-qPCR-F	GGTTCTGCCTTCGCGCTACTTC
	GmChlI1a-qPCR-R	ATTGGTAACCGAGAGCTGAGCC
GmChlI1b	GmChlI1b-qPCR-F	GGTTCTTCCTTCGCGCTGCATC
	GmChlI1b-qPCR-R	ATTGGCAACTGAGAGCTGAGAC
GmChlI2a	GmChlI2a-qPCR-F	CTTCCTCTCTTCACGATACCAC
	GmChlI2a-qPCR-R	AACACTTGTAACCTTGACGCCG
GmChlI2b	GmChlI2b-qPCR-F	CTTCCACGTTTGGCGCATCTTC
	GmChlI2b-qPCR-R	CACTCGCAACCGGGAACAGAGA
GmChlD1	GmChlD1-qPCR-F	TGAAGTGGTAGAAGGTTCCATTGC
	GmChlD1-qPCR-R	AAGACCATCTTCCCACTCTTCTGG
GmChlD2	GmChlD2-qPCR-F	GCTGCTGTTGTAGGCCAAGATG
	GmChlD2-qPCR-R	TCCTCCAATCCCAGGGTCAATG
GmChlH1	GmChlH1-qPCR-F	CATATCAGTCCCTCAAAGACACCGG
	GmChlH1-qPCR-R	CGTCTGGCAGAGTCACATCCTTGTC
GmChlH2	GmChlH2-qPCR-F	CTTATCAGTCCCTCAAAGACACAGG
	GmChlH2-qPCR-R	AGAGGGATCTCCTCACCCTCATTTG
GmChlH3	GmChlH3-qPCR-F	AATGCTCAGGTGCGTACCCTTG
	GmChlH3-qPCR-R	AGCATGCCTTCATACCACTTGGG

Supplementary Table S1 (continued). PCR primers used in this study.

Description	Primer Name	Sequence (5'→3')
**Subcellular localization**		
GmChlI1a	GmChlI1a-XbaI-F	GCATCTCTAGAATGGCGTCCACGTTGGGCACTTCTT
	GmChlI1a-XhoI-R	CGAGCCCTCGAGGCTGAATACCTCATAAAATTTCTC
GmChlI1b	GmChlI1b-XbaI-F	GCATCTCTAGAATGGCGTCCACGTTGGGCACTTCTT
	GmChlI1b-XhoI-R	CGAGCCCTCGAGGCTAAATACCTCATAAAATTTCTC
GmChlI2a	GmChlI2a-XbaI-F	GCATCTCTAGAATGGCGTCCATGTTTGGCACATCTTC
	GmChlI2a-XhoI-R	CGAGCCCTCGAGTCTGAAAACCTCGTAAAATTTCTC
GmChlI2b	GmChlI2b-XbaI-F	GCATCTCTAGAATGGCTTCCACGTTTGGCGCATCTTC
	GmChlI2b-XhoI-R	CGAGCCCTCGAGTCTGAAAACCTCATAAAATTTCTC
GmChlD1	GmChlD1-NheI-F	GCATCGCTAGCATGGGTTTCGCTTTGGCATACACAG
	GmChlD1-XhoI-R	CGAGCCCTCGAGTGAACTTTTCAAAGCTGATAAAG
GmChlD2	GmChlD2-NheI-F	GCATCGCTAGCATGGGTTTCGCTTTGGCATTCACAG
	GmChlD2-XhoI-R	CGAGCCCTCGAGTGAACTTTTCAAAGCTGATAAAG
GmChlH1	GmChlH1-NheI-F	CGCATCGCTAGCATGGCTTCTTTAGTATCTTCACAA
	GmChlH1-XhoI-R	CTAGACTCGAGGGAAGACAAAATTGAAGGCATTG
GmChlH2	GmChlH2-NheI-F	CGCATCGCTAGCATGGCTTCTTTGGTATCTTCACC
	GmChlH2-XhoI-R	CTAGACTCGAGGCGATCAATGCCTTCAATTTTGTC
GmChlH3	GmChlH3-XbaI-F	CGCATCTCTAGAATGGCTTCTTTAGTGTCTTCTCCA
	GmChlH2-XhoI-R	CTAGACTCGAGGCGATCAATGCCTTCTATTTTGTC
**Yeast two hybrid**		
GmChlI1a	GmChlI1a-NcoI-F	GCATCCCATGGAGTTGCCACTGAAGTTAACTCTG
	GmChlI1a-XhoI-R-TGA	GCGTCCTCGAGTCAGCTGAATACCTCATAA
GmChlI1b	GmChlI1b-NcoI-F	GCATCCCATGGAGTCTCAGCTCTCAGTTGCCAA
	GmChlI1b-XhoI-R-TGA	GCGTCCTCGAGTTGCCACTGAAGTTAACTCTG
GmChlI2a	GmChlI2a-NcoI-F	GCATCCCATGGTTGCCACTCAACTTAACTCTGC
	GmChlI2a-XhoI-R-TGA	GCGTCCTCGAGTCATCTGAAAACCTCGTAA
GmChlI2b	GmChlI2b-NcoI-F	GCATCCCATGGAGCTTGCCACTCAACTTAACTCTGC
	GmChlI2b-XhoI-R-TGA	GCGTCCTCGAGTCATCTGAAAACCTCATAAAATT
GmChlD1	GmChlD1-NdeI-F	CAATCGCATATGGCTCAGTCTGAAAATGGAGCTCTGG
	GmChlD1-XhoI-R-TGA	GCGTCCTCGAGTCATGAACTTTTCAAAGCTGATAA
GmChlD2	GmChlD2-NdeI-F	CAATCGCATATGGCTCAATCTGAAAACGGAGCTCTCG
	GmChlD2-XhoI-R-TGA	GCGTCCTCGAGTCATGAACTTTTCAAAGCTGATAA
GmChlH1	GmChlH1-SmaI-F	CTATTCCCGGGAGCTGCCATTGGCAATGGTCTATTC
	GmChlH1-XhoI-R-TGA	GCGTCCTCGAGTCAATTACCAAATTCTTTTCATTC
GmChlH2	GmChlH2-SmaI-F	CTATTCCCGGGAGCTGTCATTGGCAATGGCCTAT
	GmChlH2-XhoI-R-TGA	GCGTCCTCGAGTCATATTCATTACCAAATTCTTTTC
GmChlH3	GmChlH3-SmaI-F	CTATTCCCGGGAGCTGCTATGGGCAATGGTCTTTTC
	GmChlH3-XhoI-R-TGA	GCGTCCTCGAGTCAGCGTGTCATTATGTTCAATAAG

Supplementary Table S1 (continued). PCR primers used in this study.

Description	Primer Name	Sequence (5'→3')
**BiFC and plant transformation**		
GmChlI1a	GmChlI1a-XbaI-F	GCATCTCTAGAATGGCGTCCACGTTGGGCACTTCTTCAATTG
	GmChlI1a-KpnI-R	CTAGAGGTACCGCTGAATACCTCATAAAATTTCTC
GmChlI1b	GmChlI1b-XbaI-F	GCATCTCTAGAATGGCGTCCACGTTGGGCACTTCTTCAA
	GmChlI1b-KpnI-R	CTAGAGGTACCGCTAAATACCTCATAAAATTTCTC
GmChlI2a	GmChlI2a-XbaI-F	GCATCTCTAGAATGGCGTCCATGTTTGGCACATCTTC
	GmChlI2a-KpnI-R	CTAGAGGTACCTCTGAAAACCTCGTAAAATTTCTC
GmChlI2b	GmChlI2b-XbaI-F	GCATCTCTAGAATGGCTTCCACGTTTGGCGCATCTTC
	GmChlI2b-KpnI-R	CTAGAGGTACCTCTGAAAACCTCATAAAATTTCTC
GmChlD1	GmChlD1-NheI-F	GCATCGCTAGCATGGGTTTCGCTTTGGCATACACAGC
	GmChlD1-KpnI-R	CTAGAGGTACCTGAACTTTTCAAAGCTGATAAAG
GmChlD2	GmChlD2-NheI-F	GCATCGCTAGCATGGGTTTCGCTTTGGCATTCACAGC
	GmChlD2-KpnI-R	CTAGAGGTACCTGAACTTTTCAAAGCTGATAAAG
GmChlH1	GmChlH1-3 NheI F	CGCATCGCTAGCATGGCTTCTTTAGTATCTTCACAATTTAC
	GmChlH1-4 KpnI R	CTAGAGGTACCGGAAGACAAAATTGAAGGCATTGATCGC
GmChlH2	GmChlH2-3 NheI F	CGCATCGCTAGCATGGCTTCTTTGGTATCTTCACC
	GmChlH2-4 KpnI R	CTAGAGGTACCGCGATCAATGCCTTCAATTTTGTC
GmChlH3	GmChlH3-3 NheI F	CGCATCTCTAGAATGGCTTCTTTAGTGTCTTCTCCA
	GmChlH3-4 KpnI R	CTAGAGGTACCGCGATCAATGCCTTCTATTTTGTC
**Identified the *Arabidopsis* mutant**		
AtChlI	AT-I-LP	GTTACCATGTTTCGGTTATG
	AT-I-BP	TCGCATCTGAATTTCATAACCAATCTCGATACAC
	AT-I-RP	CTTCTGTCAAAGCCTTTTCG
AtChlD	AT-D-LP	ACCACTTGTTTACTACATCCAAGC
	AT-D-BP	ATATTGACCATCATACTCATTGC
	AT-D-RP	GTCTCCGTATCCGTATCTGATGT
AtChlH	AT-H-LP	ATCTAGACCGGATTAAACCGG
	AT-H-BP	CAAATCGAATTACATTCGGCGTTA
	AT-H-RP	TACTATCGGCAAAACCAGCAG

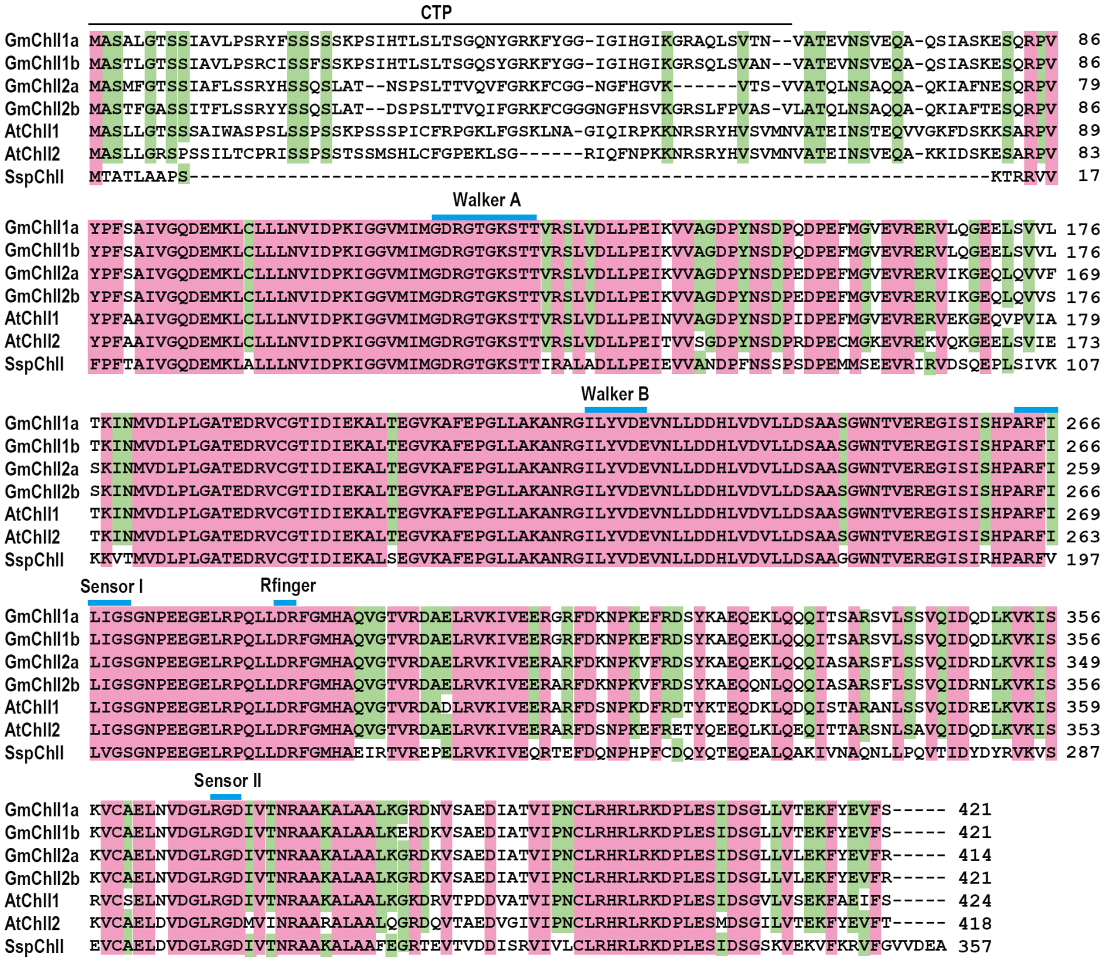

FIGURE S1. Multiple sequence alignment of Mg-chelatase I subunits.

Multiple sequence alignment analysis was performed by using ClustalW2 program. The amino acid sequences for analysis include GmChlI1a, GmChlI1b, GmChlI2a, and GmChlI2b from *Glycine max,* AtChlI1 and AtChlI2 from *Arabidopsis thaliana*, and SspChlI from cyanobacterium *Synechocystis* sp. PCC 6803. Pink and green highlight the sequences with 100% and 80% identity, respectively. The chloroplastic transit peptide (CTP) and the characteristic motifs of I subunit, including Walker A, Walker B, Sensor I, Arginine finger (Rfinger), and Sensor II, are labeled with straight lines above the cooresponding sequences.

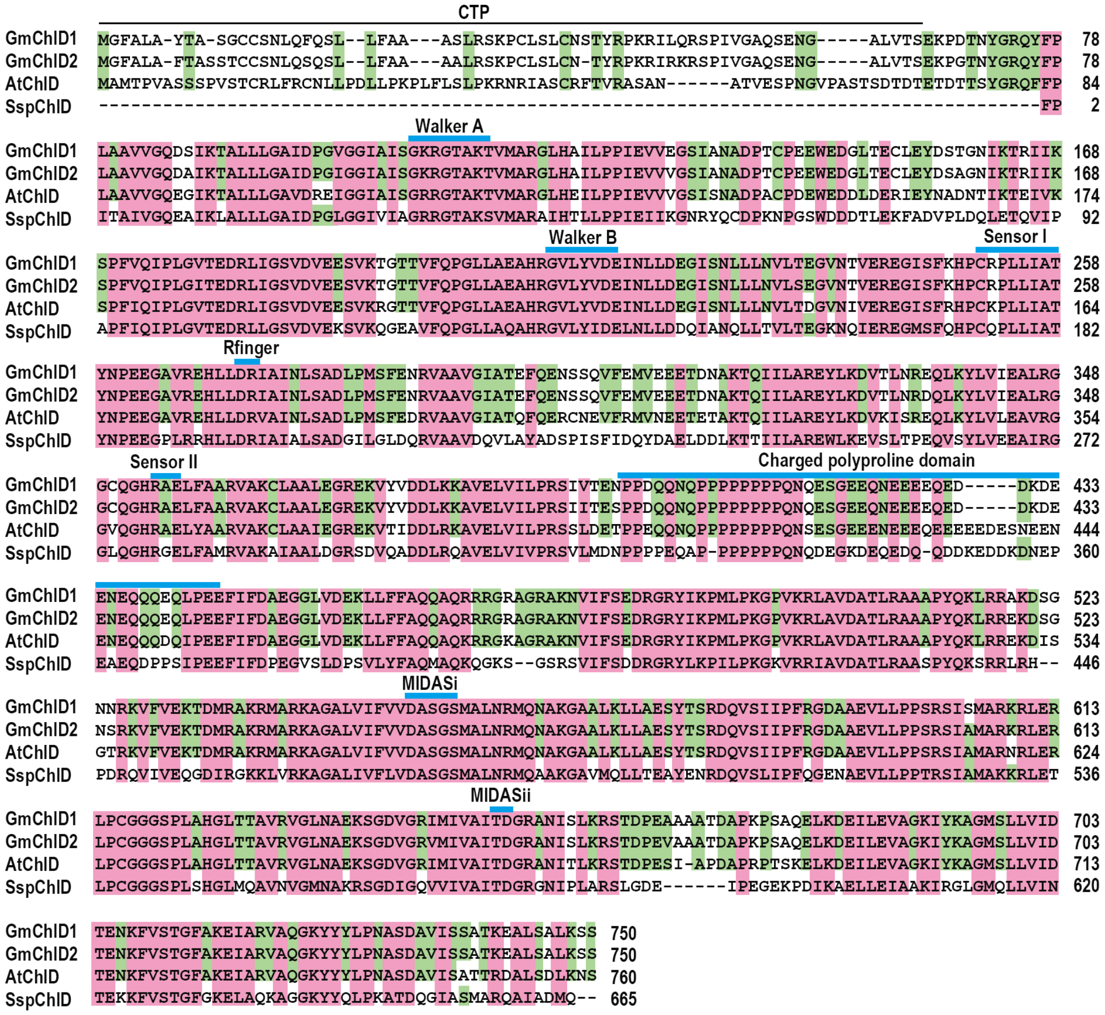

FIGURE S2. Multiple sequence alignment of Mg-chelatase D subunits.
The amino acid sequences for multiple sequence alignment analysis include GmChlD1 and GmChlD2 from *G. max*, AtChlD from *A. thaliana*, and SspChlD from Ssp. PCC 6803. Pink and green highlight the sequences with 100% and 80% identity, respectively. CTP and the characteristic motifs of D subunit, including Walker A, Walker B, Sensor I, Rfinger, Sensor II, polyproline-rich region and MIDAS motifs (MIDASi and MIDASii) are labeled above the corresponding sequences. The alignment was performed by using ClustalW2.

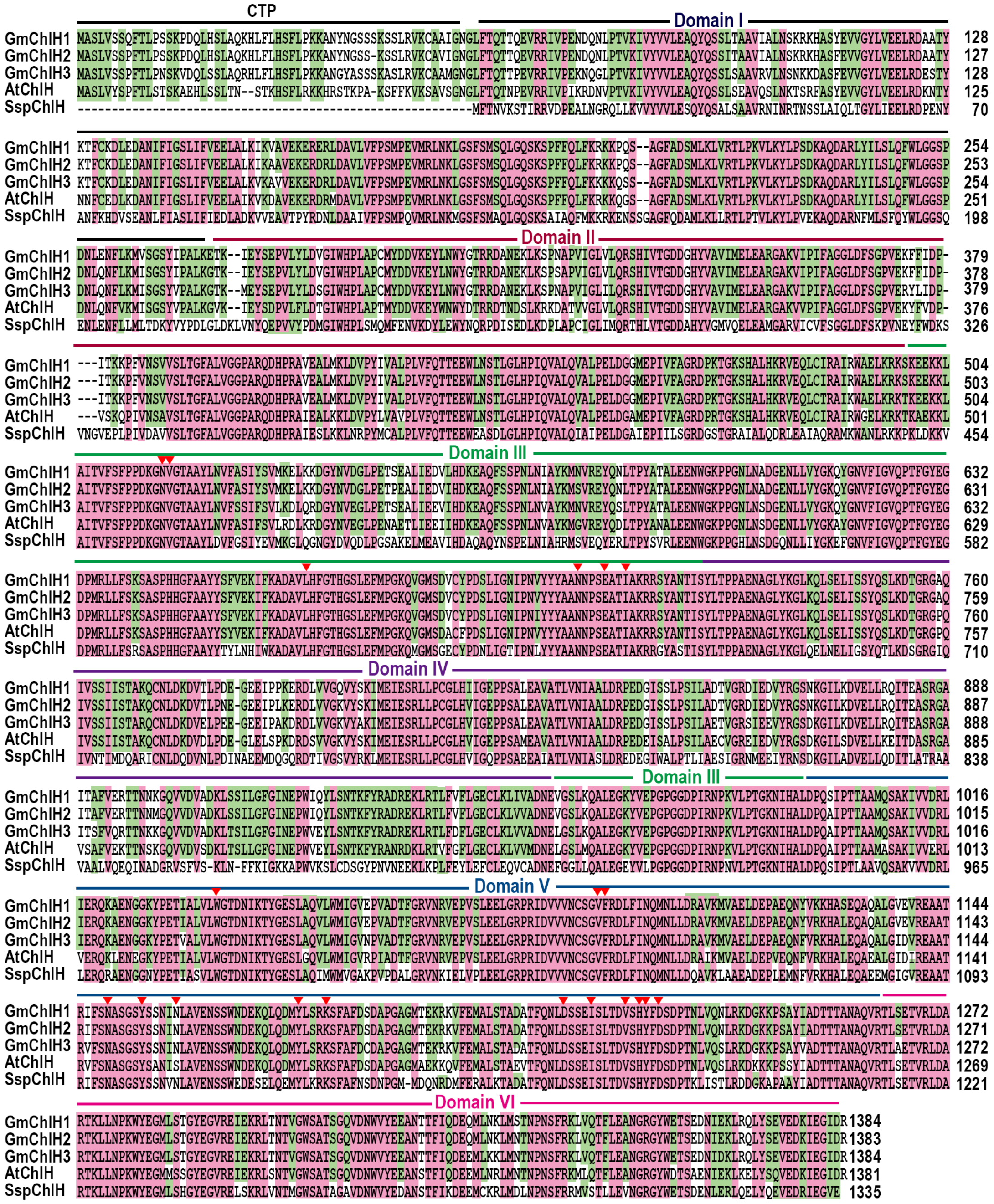

FIGURE S3. Multiple sequence alignment of Mg-chelatase H subunits.
The amino acid sequences for multiple sequence alignment analysis include GmChlH1, GmChlH2, and GmChlH3 from *G. max*, AtChlH from *A. thaliana*, and SspChlH from Ssp. PCC 6803. Pink and green highlight the sequences with 100% and 80% identity, respectively. CTP and the six structural domains (I to VI) are indicated with straight lines above the corresponding sequences. Red arrowheads point out the residues surrounding the special pocket structure located at the interface between domain III and V. This pocket possibly functions in engulfing tetrapyrrole ligand.

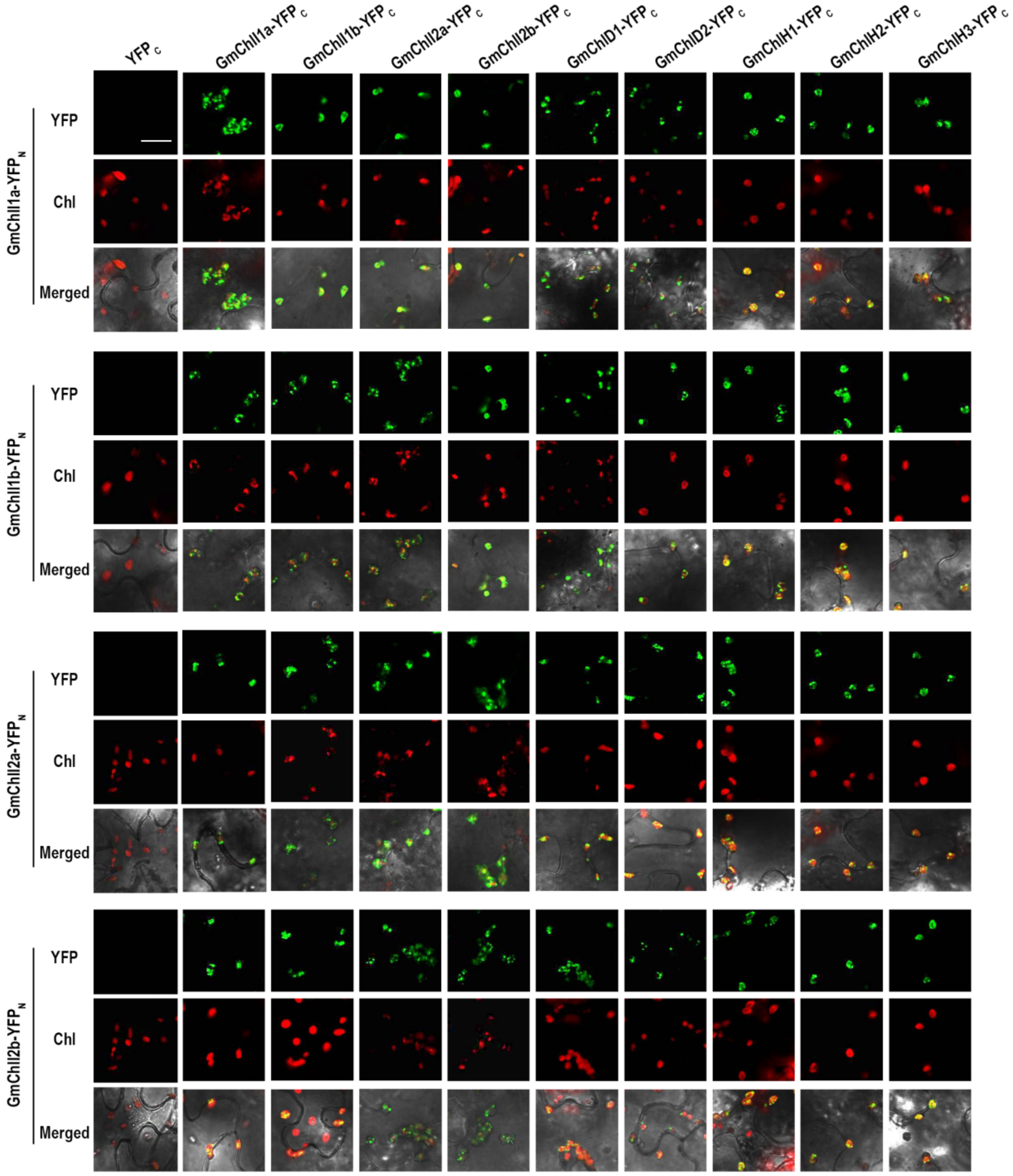

FIGURE S4. Biomolecular fluorescence complementation assay for examining the interactions of GmChlIs.

GmChlIs-YFP$_N$ were coexpressed with free YFP$_C$, GmChlIs-YFP$_C$, GmChlDs-YFP$_C$, or GmChlHs-YFP$_C$ in *N. benthamiana* leaves through *Agrobacterium* infiltration method. Leaf sectors of 3 days post infiltration (dpi) were visualized by confocal microscopy for YFP fluorescence (YFP), chlorophyll autofluorescence (Chl), and merged image of YFP, chlorophyll fluorescence and bright-field (Merged). All the images are in the same scale. Scale bar =20μm.

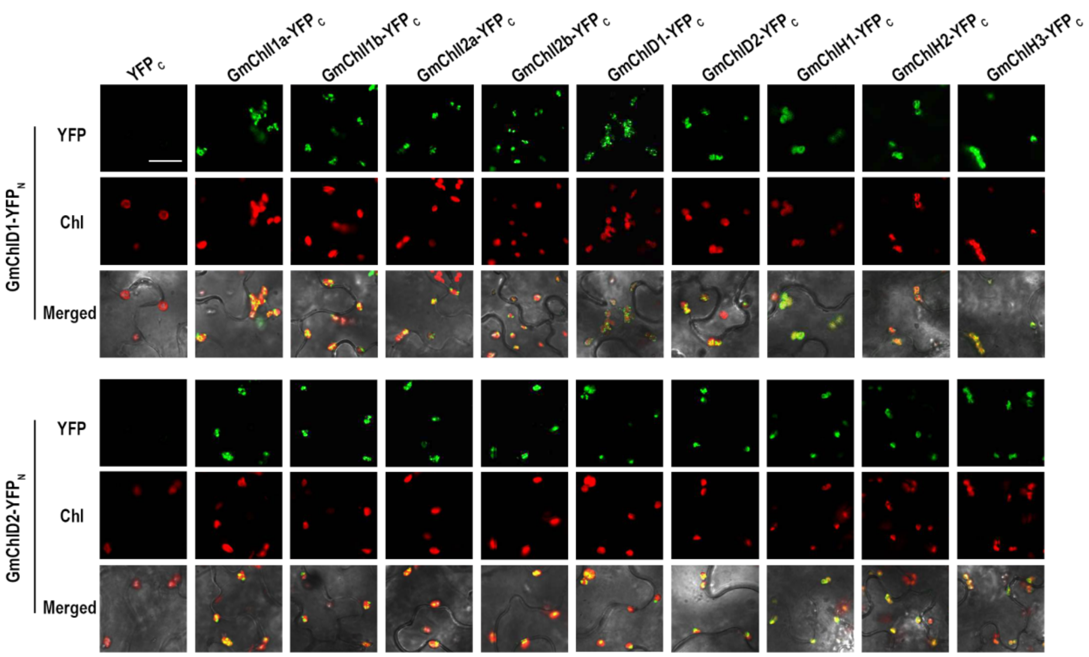

FIGURE S5. Biomolecular fluorescence complementation assay for examining the interaction of GmChlDs.

GmChlDs-YFP$_N$ were coexpressed with free YFP$_C$, GmChlIs-YFP$_C$, GmChlDs-YFP$_C$, or GmChlHs-YFP$_C$ in *N. benthamiana* leaves through *Agrobacterium* infiltration method. Leaf sectors of 3 days post infiltration (dpi) were visualized by confocal microscopy for YFP fluorescence (YFP), chlorophyll autofluorescence (Chl), and merged image of YFP, chlorophyll fluorescence and bright-field (Merged). All the images are in the same scale. Scale bar =20 μm.

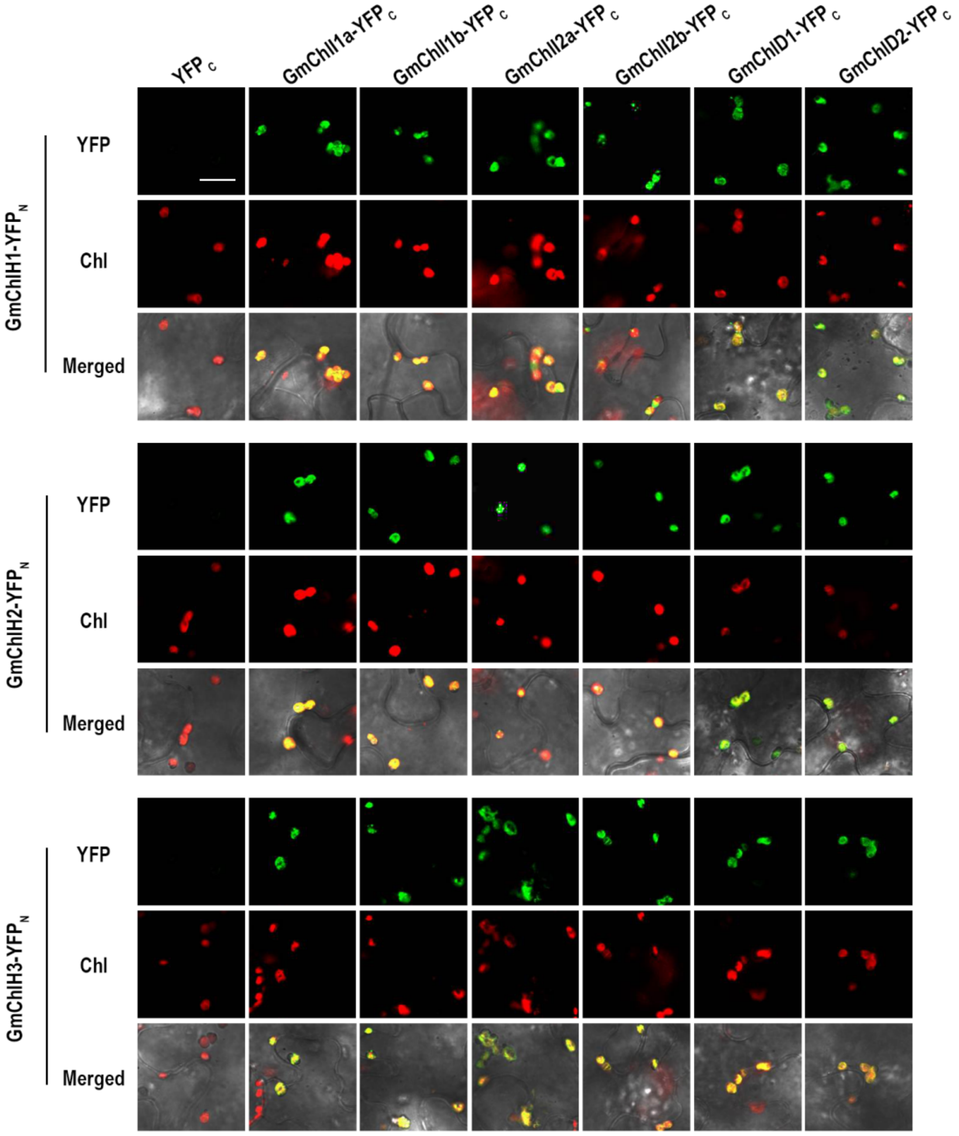

FIGURE S6. Biomolecular fluorescence complementation assay for examining the interaction of GmChlHs.

GmChlHs-YFP$_N$ were coexpressed with free YFP$_C$, GmChlIs-YFP$_C$, GmChlDs-YFP$_C$ or GmChlHs-YFP$_C$ in *N. benthamiana* leaves through *Agrobacterium* infiltration method. Leaf sectors of 3 days post infiltration (dpi) were visualized by confocal microscopy for YFP fluorescence (YFP), chlorophyll autofluorescence (Chl), and merged image of YFP, chlorophyll fluorescence and bright-field (Merged). All the images are in the same scale. Scale bar =20 μm.

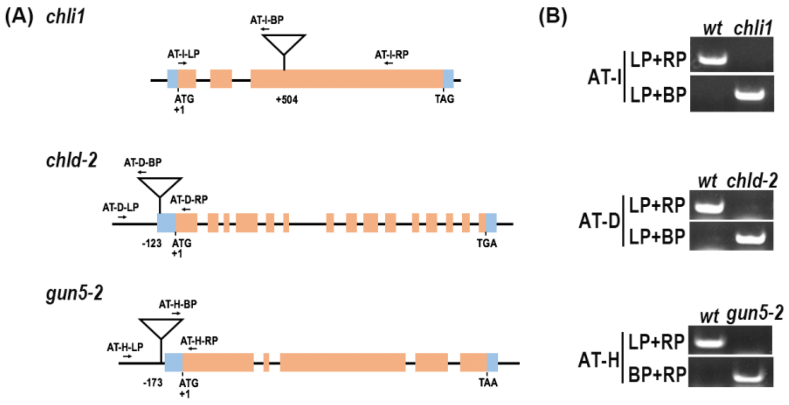

FIGURE S7. Identification of *chli1*, *chld-2* and *gun5-2* mutants.
(A) Schematic representation of T-DNA insertion sites in *chli1*, *chld-2* and *gun5-2*. Exons are shown as orange boxes, 5' and 3' UTRs as blue boxes, and introns as black lines. The position of the T-DNA insertion site is indicated with respect to the translation initiation site. Primers used to identify homozygous mutants are indicated by arrows above corresponding positions. The T-DNA insert is not drawn to scale; **(B)** Genotyping of mutants performed via PCR using the primers shown in (A).
